# Malaria in pregnancy in India: a 50-year bird’s eye

**DOI:** 10.3389/fpubh.2023.1150466

**Published:** 2023-10-19

**Authors:** Loick Pradel Kojom Foko, Vineeta Singh

**Affiliations:** Parasite and Host Biology Group, ICMR-National Institute of Malaria Research, New Delhi, India

**Keywords:** malaria, pregnancy, epidemiology, outcomes, prevention, treatment, India

## Abstract

**Introduction:**

In 2021, India contributed for ~79% of malaria cases and ~ 83% of deaths in the South East Asia region. Here, we systematically and critically analyzed data published on malaria in pregnancy (MiP) in India.

**Methods:**

Epidemiological, clinical, parasitological, preventive and therapeutic aspects of MiP and its consequences on both mother and child were reviewed and critically analyzed. Knowledge gaps and solution ways are also presented and discussed. Several electronic databases including Google scholar, Google, PubMed, Scopus, Wiley Online library, the Malaria in Pregnancy Consortium library, the World Malaria Report, The WHO regional websites, and ClinicalTrials.gov were used to identify articles dealing with MiP in India. The archives of local scientific associations/journals and website of national programs were also consulted.

**Results:**

Malaria in pregnancy is mainly due to *Plasmodium falciparum* (*Pf*) and *P. vivax* (*Pv*), and on rare occasions to *P. ovale* spp. and *P. malariae* too. The overall prevalence of MiP is ~0.1–57.7% for peripheral malaria and ~ 0–29.3% for placental malaria. Peripheral *Pf* infection at antenatal care (ANC) visits decreased from ~13% in 1991 to ~7% in 1995–1996 in Madhya Pradesh, while placental *Pf* infection at delivery unit slightly decreased from ~1.5% in 2006–2007 to ~1% in 2012–2015 in Jharkhand. In contrast, the prevalence of peripheral *Pv* infection at ANC increased from ~1% in 2006–2007 to ~5% in 2015 in Jharkhand, and from ~0.5% in 1984–1985 to ~1.5% in 2007–2008 in Chhattisgarh. Clinical presentation of MiP is diverse ranging from asymptomatic carriage of parasites to severe malaria, and associated with comorbidities and concurrent infections such as malnutrition, COVID-19, dengue, and cardiovascular disorders. Severe anemia, cerebral malaria, severe thrombocytopenia, and hypoglycemia are commonly seen in severe MiP, and are strongly associated with tragic consequences such as abortion and stillbirth. Congenital malaria is seen at prevalence of ~0–12.9%. Infected babies are generally small-for-gestational age, premature with low birthweight, and suffer mainly from anemia, thrombocytopenia, leucopenia and clinical jaundice. Main challenges and knowledge gaps to MiP control included diagnosis, relapsing malaria, mixed *Plasmodium* infection treatment, self-medication, low density infections and utility of artemisinin-based combination therapies.

**Conclusion:**

All taken together, the findings could be immensely helpful to control MiP in malaria endemic areas.

## 1. Introduction

Globally, an estimated 247 million cases and 619,000 deaths were due to malaria in 2021 (1). This burden has significantly increased compared to previous years, partially due to the current COVID-19 pandemics (2, 3). Malaria is due to five *Plasmodium* species that are transmitted to human through infecting bites of female *Anopheles* mosquitoes (4). *Plasmodium falciparum* (*Pf*) and *Plasmodium vivax* (*Pv*) are the predominant malaria species around the world; *Pf* is the most severe and dangerous species while *Pv* is the most geographically spread but can also induce severe clinical attacks (5, 6).

The sub-Saharan Africa (sSA) and South East Asia (SEA) regions are affected by malaria, especially children under 5 years of age and pregnant women (2). Malaria during pregnancy (MiP) poses an important problem to both; the future mother and unborn child. During pregnancy, malaria infection is associated with several maternal, fetal and birth complications including growth restriction, stillbirth, premature delivery, spontaneous abortions, low birth weight (LBW), and even death of mother and/or child (7, 8). The clinical spectrum and outcomes of MiP are various with distinct features depending on factors such as epidemiological situation of the setting, malaria species, gravidity, and coverage of malaria control measures [e.g., intermittent preventive treatment with sulfadoxine + pyrimethamine – IPTp-SP, indoor residual spraying (IRS), and long lasting insecticide-treated nets (LLINs)] (2).

India accounted for ~79% of malaria cases and ~ 83% of deaths seen in SEA in 2021 (2). In the present review, we reviewed the situation of MiP in India with emphasis on its epidemiology, clinical presentation, determinants, outcomes, prevention, and treatment. A brief overview of neonatal and congenital malaria (NCM) characteristics is also presented. Finally, we identify knowledge gaps on MiP research and propose solutions for future directions.

## 2. Materials and methods

### 2.1. Search strategy

The strategy used to identify relevant studies was inspired from MiP reviews reported previously (7, 9). Briefly, we used Google scholar, Google, PubMed, Scopus, Wiley Online library, the Malaria in Pregnancy Consortium library, the World Malaria Report, The WHO regional websites, and ClinicalTrials.gov to search for articles dealing with MiP in India published in last 50 years. The archives of local scientific associations/journals (e.g., Indian Journal of Medical Research, Indian Journal of Malariology now known as Journal of Vector Borne Diseases) and websites of national programs were also consulted. We used the search terms “malaria,” “pregnancy,” “pregnant woman,” “burden,” “prevalence,” “epidemiology,” “outcome,” “placental infection,” “congenital malaria,” “neonatal malaria,” “diagnostic,” “prevention,” “control,” “management,” “India,” and Indian regions (Tamil Nadu, Chandigarh, Andaman and Nicobar, Assam, Andhra Pradesh, Bihar, Chhattisgarh, Daman and Diu, Goa, Delhi, Gujarat, Himachal Pradesh, Jammu and Kashmir, Jharkhand, Kerala, Kolkata, Karnataka, Lakshadweep, Maharashtra, Manipur, Mizoram, Madhya Pradesh, Meghalaya, Nagaland, Odisha, Pondicherry, Rajasthan, Sikkim, Tripura, Uttarakhand, Uttar Pradesh, Punjab, Haryana, and West Bengal). Boolean operators including “AND” and “OR” were used in combination with the above mentioned search terms to identify relevant papers through databases such as PubMed. The search strategy was tailored to each of the search databases using search terms and Boolean operators (AND, OR). We also included all publications on neonatal and congenital malaria (NCM). To do so, the same search strategy used for MiP papers was used with some differences. For example search terms for NCM papers were “malaria,” “placental infection,” “congenital malaria,” “neonatal malaria,” “India,” and Indian regions (Tamil Nadu, Chandigarh, Andaman and Nicobar, Assam, Andhra Pradesh, Bihar, Chhattisgarh, Daman and Diu, Goa, Delhi, Gujarat, Himachal Pradesh, Jammu and Kashmir, Jharkhand, Kerala, Kolkata, Karnataka, Lakshadweep, Maharashtra, Manipur, Mizoram, Madhya Pradesh, Meghalaya, Nagaland, Odisha, Pondicherry, Rajasthan, Sikkim, Tripura, Uttarakhand, Uttar Pradesh, Punjab, Haryana, and West Bengal).

### 2.2. Screening strategy

Titles and abstracts of studies retrieved from databases were independently reviewed by the authors in order to identify those relevant to the study. The full texts were retrieved and scrutinized to extract data of interest. Principal investigators were kindly contacted to request full length paper and/or more details on studies. We also contacted editors-in-chief of national journals to request full length papers in case of a negative reply or no reply at all from principal investigators. Additionally, we reviewed relevant articles cited in references of identified literature and included them as primary sources.

### 2.3. Eligibility criteria

Only papers published in English and Hindi were included. Publications were considered of interest if they addressed any aspect of MiP and NCM in India including prevalence, clinical presentation, determinants, maternal and fetal/neonatal outcomes, diagnostic, prevention, and treatment. The list of studies is presented in Supplementary Tables 1–5.

### 2.4. Data extraction

Data of interest were independently extracted from eligible publications, and these consisted of (i) characteristics of studies (first author’ name, year of publication, study design, area, state/union territory, urbanization setting, and year of sample collection); (ii) demographical, obstetrical, and gynecological data (age, timing of screening, parity, trimester of gestation, and route of delivery); (iii) clinical characteristics of MiP and NCM (type of malaria, clinical signs/symptoms, presence of comorbidities, and levels of hemoglobin, blood cells, and biochemical markers); (iv) parasitological information (blood source, parasitological screening method, malaria species, total number of individuals included, total number of malaria infected individuals, number of mono-infections for each malaria species, and number of mixed infections), (v) factors associated with malaria infection in pregnant women and babies; (vi) MiP and NCM outcomes, (vii) malaria preventive methods used, and (viii) efficacy of treatments to control MiP. Setting urbanization was categorized as urban, semi-urban, rural, and not specified. Timing of screening consisted of women screened for malaria parasites in community or at health facility for delivery (DU) and antenatal care visit (ANC). Type of malaria was defined either as asymptomatic malaria, uncomplicated malaria (UM) or severe malaria (SM). In the early 90s, the WHO defined a set of criteria used for diagnosing severe malaria (SM) in children and adults (Table 1) (6, 10, 12–16). The latest WHO guideline lists 12 SM-associated signs/symptoms by malarial species and age group: severe malarial anemia (SMA), severe renal impairment, cerebral malaria (CM; prostration, impaired consciousness/coma, and multiple convulsions), jaundice, hypoglycemia, acidosis/acute respiratory distress syndrome (ARDS), significant bleeding, pulmonary edema, circulatory collapse/shock, and hyperparasitemia (10).

**Table 1:** Evolution of WHO definitions of severe malaria clinical and laboratory manifestations due to *Plasmodium falciparum* (1990–2015), *Plasmodium vivax* (2006–2015), and *Plasmodium knowlesi* (2012–2015) in children, non-pregnant adults, and pregnant women (10–16).

Signs/symptoms	Definitions	1990	2000	2006	2010	2012	2014	2015
Severe anemia^*,&^	Hemoglobin (Hb) < 5 g/dL, or Hematocrit (Hct) < 15%							
	Hb < 5 g/dL, or Hct < 15% (Children) Hb < 7 g/dL, or Hct < 20% (Adults)							
	Hb < 5 g/dL, or Hct < 15% (Children <12 years) with parasitemia >10,000 p/μL Hb < 7 g/dL, or Hct < 20% (Adults) with parasitemia >10,000 p/μL							
Severe renal impairment^*^	Urine output <12 mL/kg/24 h, or plasma creatinine concentration above the age-related normal values (Children)/Urine output <400 mL/kg/24 h, or/and a serum creatinine >3 mg/dL (Adult), and despite adequate volume repletion/rehydration							
	Serum creatinine concentration > 265 μmol/L (3 mg/dL)							
	Serum creatinine concentration > 265 μmol/L (3 mg/dL) or blood urea >20 mmol							
Shock/Circulatory collapse^*^	Systolic blood pressure (SBP) < 70 mmHg (Adults) and < 50 mmHg (Children 1–5 years)							
	SBP < 80 mmHg (Adults) and < 50 mmHg (Children)							
	*Compensated shock*: capillary refill ≥3 s or temperature gradient on leg (mid to proximal limb), but no hypotension. *Decompensated shock*: SBP < 70 mmHg (Children) or < 80 mmHg (Adults) with confirmation of impaired perfusion (prolonged capillary refill or cool peripheries)							
Abnormal bleeding^*^	Spontaneous bleeding from gums, nose, gastrointestinal tract, and venipuncture sites. Clinical evidence of bleeding using tests (e.g., prothrombin time, platelet)							
	Recurrent or prolonged bleeding from nose gums, gastro-intestinal tract, or venipuncture sites; hematemesis or melena							
Disseminated intravascular coagulation (DIC)	Laboratory evidence (e.g., prothrombin time prolonged *>*3 s of the control)							
Multiple convulsions	More than 2 convulsions observed within 24 h							
Metabolic acidosis^*^	Plasma bicarbonate <15 mmol/L							
	Plasma bicarbonate <15 mmol/L or base excess (≥ 10 mmol/L)							
	A base deficit of >8 mEq/L or, if unavailable, a plasma bicarbonate of <15 mM or venous plasma lactate >5 mM							
Hemoglobinuria^*^	Hemolysis not secondary to glucose-6-phosphate dehydrogenase deficiency							
	Urine is dark or black, and urinalysis dipstick test is positive for Hb, associated with absence of microscopic hematuria (i.e., presence of blood in urine)							
Impaired consciousness/Cerebral malaria^*,§^	Rousable coma (impaired consciousness)/Unarousable come (cerebral malaria)							
	A Glasgow coma Score < 9 (Adults), or a Blantyre coma score < 2 (Children)							
	A Glasgow coma Score < 11 (Adults), or a Blantyre coma score < 3 (Children)							
Prostration^*^	Weakness so that the patient cannot sit or walk, with no obvious neurological explanation							
	The inability to sit upright or to drink (Children), Extreme weakness (Adults)							
	Generalized weakness so that the patient is unable walk or sit up without assistance							
Clinical jaundice^*^	Plasma/serum bilirubin concentration > 50 μmol/L (3 mg/dL)^a^							
	Plasma/serum bilirubin concentration > 50 μmol/L (3 mg/dL)^b^							
	Plasma/serum bilirubin concentration > 50 μmol/L (3 mg/dL) with parasite density > 20,000 parasites/μL^c^							
	Plasma/serum bilirubin concentration > 50 μmol/L (3 mg/dL) with parasite density > 100,000 parasites/μL^a,c^							
Multi-organ dysfunction	Clinical jaundice, and evidence of other vital organ dysfunction							
Pulmonary oedema^*^	Diagnosed upon radiological examination							
	Diagnosed upon radiological examination, or oxygen saturation < 92% on room air with a respiratory rate > 30/min, often with chest indrawing and crepitations on auscultation							
Acidotic breathing	Deep breathing and respiratory distress							
Hypoglycaemia	Whole blood glucose concentration < 2.2 mmol/L (40 mg/dL)							
Hyperpyrexia	Core body temperature > 40°C							
Hyperlactatemia^*^	Plasma lactate 5 mmol/L (Children) and > 6 mmol/L (Adults)							
	Plasma lactate >5 mmol/L							
Failure to feed	n.a (observable)							
Hyperparasitemia^*^	No parasitemia threshold defined^a^							
	Parasitemia >4% (unstable malaria endemicity), > 20% (stable malaria endemicity)^a^							
	Parasitemia >5% (low malaria endemicity), > 10% (high malaria endemicity)^a^							
	Parasitemia >2% (low malaria endemicity), > 5% (high malaria endemicity)^a^							
	Parasitemia >20% in any epidemiological context^a^							
	Parasitemia >10% in any epidemiological context^a^							
	Parasitemia >2%^c^							

DIC, Disseminated intravascular coagulation; Hb, Hemoglobin; Hct, Hematocrit; n.a, Not applicable; *Pf*, *Plasmodium falciparum*; *Pk*, *Plasmodium knowlesi*; *Pv*, *Plasmodium vivax*; SBP, Systolic blood pressure; WHO, World Health Organization. ^*^These signs/symptoms have been revised by the WHO. ^&^Defined as severe normocytic anemia in the previous guidelines (1990–2012). ^§^Impaired consciousness (rousable coma) and cerebral malaria (unarousable coma) were clinically differentiated before advent of coma scales (16). *Pv* and *Pk* have been first mentioned as able to induce severe malaria in the 2006 and 2012 WHO guidelines, respectively. Manifestations of severe malaria due to *Pv* parasites may present to with some symptoms similar to severe *Pf* malaria. Manifestations of severe malaria due to *Pk* parasites are similar to those of severe *Pf* malaria with the exception of coma. ^a^Used for *P. falciparum* severe malaria only. ^b^Used for *P. vivax* severe malaria only. ^c^Used for P. knowlesi severe malaria only. The color is related to linking the definition of a given sign/symptom to the period where this definition was retained in global WHO guidelines to diagnose severe malaria.

### 2.5. Data management

Data were keyed into an Excel spreadsheet (Microsoft Office, United States) by reviewer authors and then coded and verified for consistency, and removed any duplicates. Any discrepancies between the authors were resolved through discussion and consensus. Data were analyzed using StatView 5.0 for Windows (SAS Institute, Inc., Chicago, Illinois, United States) and GraphPad v8.01 for Windows (GraphPad, Inc., California, United States), and summarized as percentages and mean in tables and charts where appropriate. Overall aggregation of data using sophisticated approaches such as meta-analysis was not possible due to the heterogeneity of studies related to study design, diagnostic methods, timing of screening, blood origin, analysis conducted, and effect measures presented. Thus, only summaries of study findings stratified by variables such as diagnostic methods, timing of screening and blood origin are presented in this review. In addition, findings from studies with minimum sample size of 30 were extracted to generate charts (17). We think that this approach of analysis is more appropriate to provide reliable results and avoid misleading conclusions on Indian scenario of MiP and NCM.

## 3. Results

### 3.1. Burden of malaria in India

**Figure 1:**
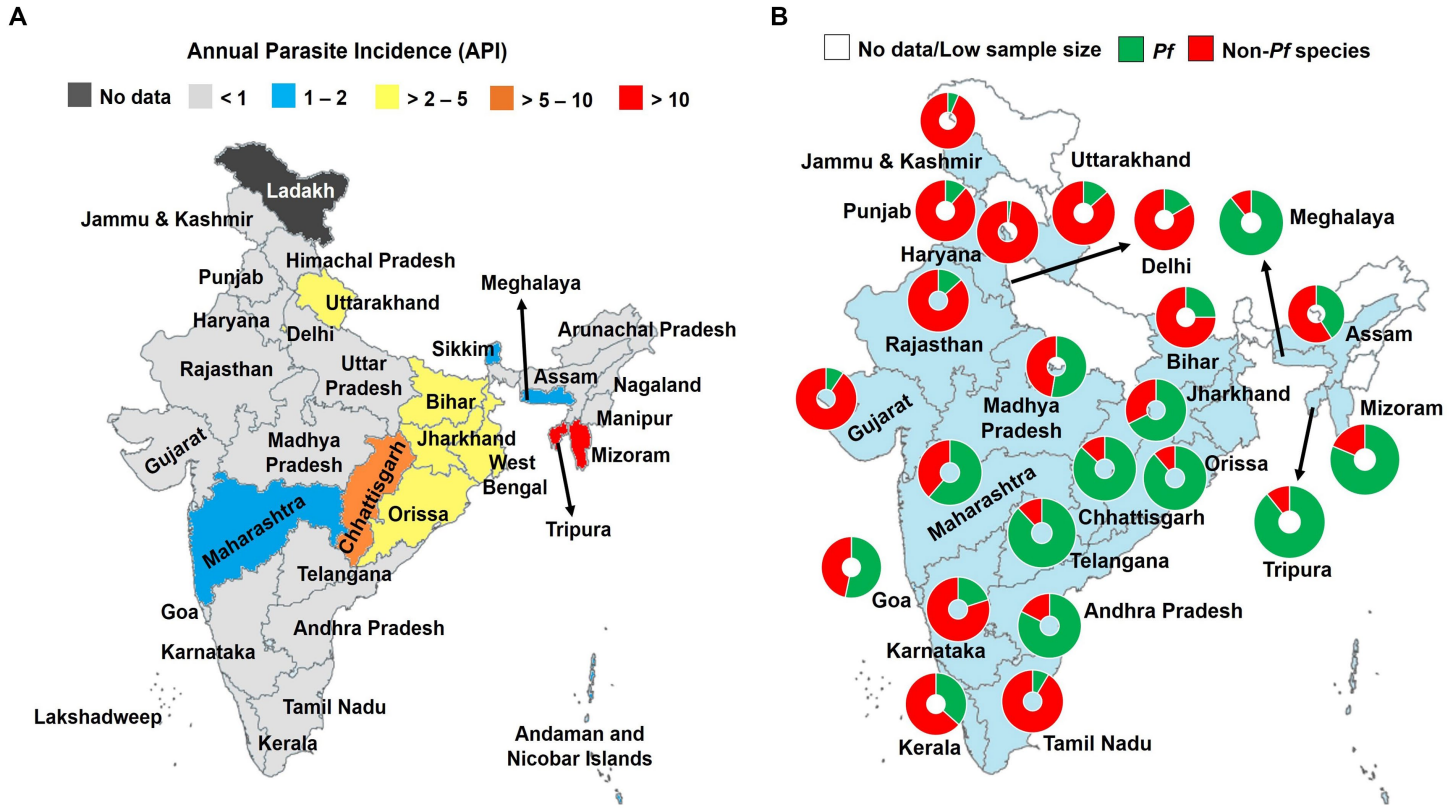
Burden of malaria in India, 2021. **(A)** Annual parasite incidence of *Plasmodium* spp. infections, and **(B)** contribution of *Pf* and non-*Pf* species. API, Annual parasite incidence; *Pf*, *Plasmodium falciparum*; *Pv*, *P. vivax*. Malaria species were identified using light microscopy Non-*Pf* species are mostly represented by *Pv* (>90%). The data were retrieved from official website of the National Vector Borne Disease Control Program (https://nvbdcp.gov.in). Pie charts depict the relative contribution of malaria species in total malaria cases. Only sample size of malaria positive slides >30 were retained and presented in **(B)** (17). The map was retrieved from the official website of the Ministry of External Affairs of Government of India (https://mea.gov.in/india-at-glance.htm).

Malaria burden profoundly decreased in last two decades in India, and this is due to diverse strategies implemented and/or scaled up over the country (e.g., LLINs, IRS) (6). The recent national data from National Vector Borne Disease Control Program (NVBDCP) indicated that malaria transmission is low with annual parasite incidence (API) < 1 in most of the areas of India (https://nvbdcp.gov.in; Figure 1A). Malaria control needs to be reinforced in some areas including Bihar, Delhi, Uttarakhand, Orissa, Chhattisgarh, and West Bengal where API is >2 (Figure 1A). The risk of malaria infection is highest in two North-Eastern areas of India namely Tripura and Mizoram with API > 10.

The *Pf* and *Pv* species are major species in the country, with ratio close to one at the country level but varied between the different regions (Figure 1B) (18–20). Other species including *P. ovale* (*Po*), *P. malariae* (*Pm*), and *P. knowlesi* (*Pk*) are also found in India but much fewer in extent than that of *Pf* and *Pv* (21–24). The *Pm* species was reported from Madhya Pradesh, Andhra Pradesh, Tamil Nadu, Kerala, Karnataka, and Orissa; while *Po* spp. was mainly seen in Uttar Pradesh, Assam, and Gujarat states (21, 23). Tyagi and colleagues reported the circulation of *Pk* in patients living in the Andaman and Nicobar Islands (24). Recently, *Pk* was reported from Bihar, Uttar Pradesh, and Delhi (22).

### 3.2. Burden of MiP in India

**Figure 2:**
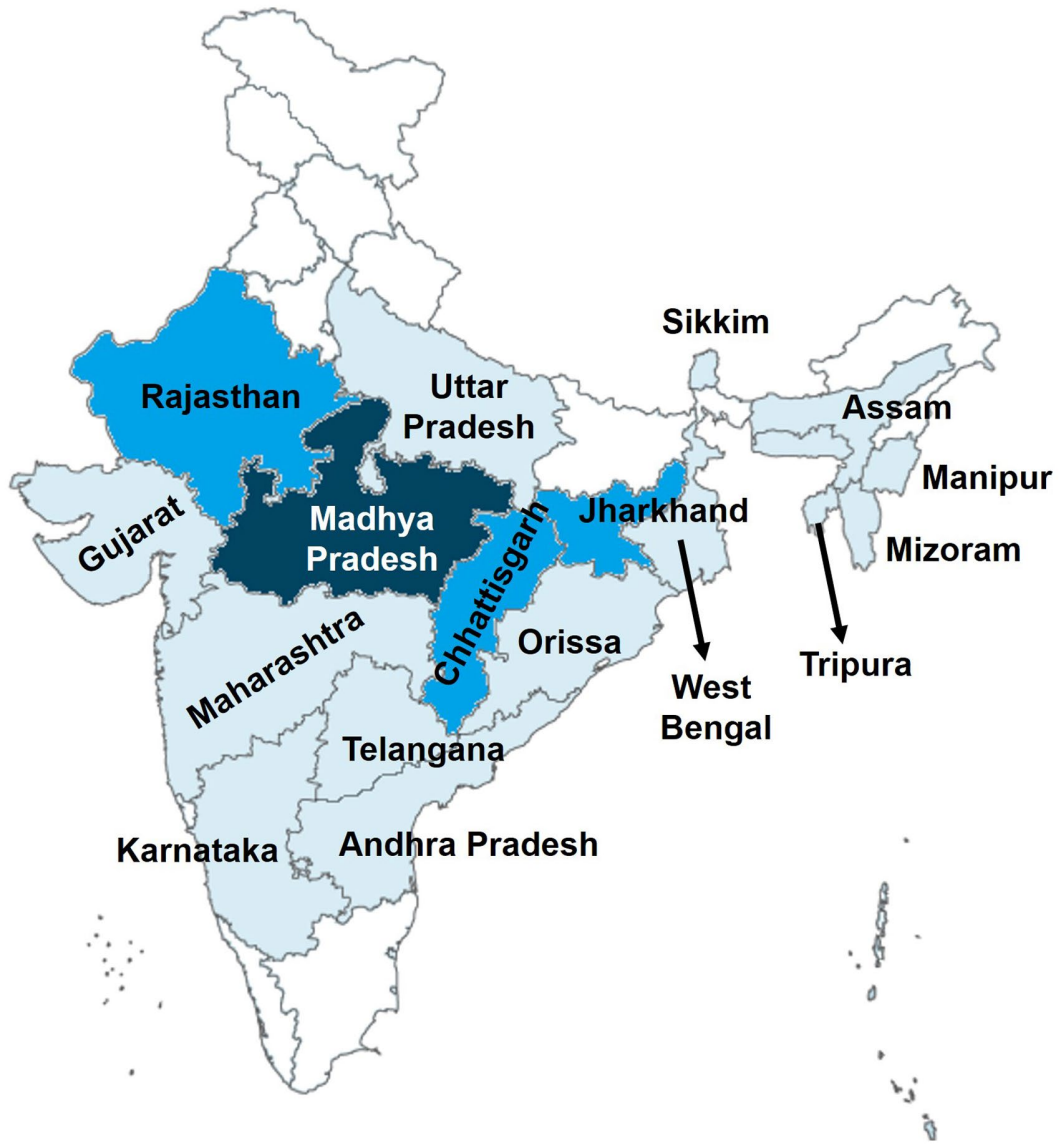
Geographical distribution of studies on MiP in India. The map was retrieved from official website of the Ministry of External Affairs of Government of India (https://mea.gov.in/india-at-glance.htm). Areas colored represent the places where studies on MiP were conducted. The darker the area higher is the number of conducted studies on MiP in those areas.

Data on the epidemiology of MiP in India are largely heterogeneous due to variation in diagnostic methods (LM, PCR, RDT, and histology), timing of screening (ANC, DU, and community), and blood origin (peripheral and placental blood). Again, results are more documented from forested, tribal, and rural areas of three states *viz.* Madhya Pradesh, Rajasthan, and Chhattisgarh (Table 2; Figure 2). Information on MiP epidemiology is lacking in highly malaria prevalent areas such as Delhi, Bihar, and some North Eastern states (e.g., Arunachal Pradesh and Nagaland). On ANC visits, prevalence of MiP in Madhya Pradesh state ranged from 1.9 to 17.9% using LM for detecting malaria parasites in the peripheral blood (Table 2). In Chhattisgarh, peripheral blood-based MiP prevalence ranged from 20.6 and 29.3% using RDT (42, 49). Combining different diagnostic methods, several authors reported placental malaria prevalence at DU of 2.2 and 29.3% in Madhya Pradesh (35, 40), and 21.9% in Uttar Pradesh (26). In Madhya Pradesh and Chhattisgarh, placental malaria was diagnosed at higher rates using PCR compared to impression smear and LM (44, 47), thereby outlining a high proportion of submicroscopic infections during pregnancy. Fewer studies reported MiP burden in community where peripheral malaria prevalence rates of 55.5% using LM and 0.81% using RDT were reported in Madhya Pradesh and Chhattisgarh, respectively (33, 54).

**Table 2:** Studies conducted on burden of MiP in India.

Design	Timing of screening	States (Areas)	Setting^†^	Year of collection	Screening method	*N*	*Plasmodium* spp. infection prevalence	Ref.
Case–Control	ANC	Chandigarh and neighboring villages	Rural area	1984–1985	LM	5,589	1.40%^#^	(25)
Cross-sectional	DU	Uttar Pradesh	Not specified	Not specified	Histology/LM	256	21.9%^‡&^	(26)
Cross-sectional	DU/ANC	Gujarat (Surat)	Not specified	1987–1988	LM	Not specified	57.7%^#^	(27)
Cross-sectional	ANC	Madhya Pradesh (Jabalpur)	Rural, tribal, and urban	1991	LM	Total (831), Dry (62), Monsoon (466), and Autumn (303)	Total (17.4%^#^), Dry (19.4%^#^), Monsoon (14.8%^#^), and Autumn (21.1%^#^)	(28)
Cross-sectional	ANC	Madhya Pradesh (Jabalpur)	Rural, tribal, and urban	1991–1993	LM	1,000	20%^#^	(29)
Cross-sectional	ANC	Madhya Pradesh (Mandla District)	Rural and Forested	1995–1996	LM	456 + 325 (781)	12.9%^#^ + 11.4%^#^ (12.3%^#^)	(30)
Cross-sectional	ANC	Madhya Pradesh (Jabalpur)	Rural, tribal, and urban	1992–1995	LM	1,598	17.9%^#^	(31)
Cross-sectional	ANC	Orissa (Koraput)	Rural, tribal and with perennial hyperendemic transmission	Not specified	LM	209	11.6%^#^	(32)
Cross-sectional	-	Madhya Pradesh (Mandla District)	Rural and Forested	1997–1998	LM	274	55.1%^#^	(33)^§^
Cross-sectional	-	Madhya Pradesh (Mandla District)	Rural and Forested	1996	LM	100	30^#^	(34)^§^
Cross-sectional	DU	Madhya Pradesh (Mandla District)	Rural and Forested	2002–2003	LM/RDT	182	29.3%^‡^	(35)
Cross-sectional	DU	Madhya Pradesh (Mandla and Satna districts)	Rural, forested and tribal	2002–2003	LM	209 (Mandla), 590 (Satna)	Mandla: 5.3%^#^ and 14.4%^‡^	(36)
							Satna: 6.9%^#^ and 10.8%^‡^	
Cross-sectional	DU/ANC	Madhya Pradesh	Rural, tribal and urban	2006	LM	1,825 (ANC), 1,012 (DU)	Dry season: 1.9%^#^ (ANC), 0.8%^#^ (DU); Post-rainy season: 6.4%^#^ (ANC), 2.9%^#^ (DU)	(37)
							Dry season: 0.8%^‡^ and Post-rainy season: 2.9%^‡^	
Cross-sectional	ANC	Maharashtra (Mumbai)	Not specified	Not specified	LM	416	6.5%^#^	(38)
Cross-sectional	DU/ANC	Jharkhand (Ranchi, Konbir, and Gumla)	Rural, semi-urban, and urban	2006–2007	LM/RDT	Peripheral: 2,382 (ANC) and 717 (DU); Placental: 0 (ANC) and 712 (DU)	1.8%^#^ (ANC) and 1.7%^#^ (DU); (ANC) and 2.4%^‡^ (DU)	(39)
Cross-sectional	DU/ANC	Madhya Pradesh (Jabalpur)	Rural, tribal and urban	2008–2009	LM	500	1.8%^#^ and 2.2%^‡^	(40)
Cross-sectional	DU/ANC	Chhattisgarh (Bastar, Rajnandgaon)	Rural and Forested (Bastar is high endemic, Rajnandgaon is low endemic)	2007–2008	LM/RDT	Rajnandgaon: 1,498 (ANC), 547 (DU); Bastar: 1,198 (ANC), 481 (DU)	Total: 1.3%^#^ (ANC), 1.9%^#^ (DU)	(41)
							Rajnandgaon: 0.1%^#^ (ANC), 0.6%^#^ (DU) and 3.2%^‡^ (DU); Bastar: 2.8%^#^ (ANC), 3.4%^#^ (DU) and 3.6%^‡^ (DU)	
Cross-sectional	ANC	Chhattisgarh (Maita, Mallampeta, Dharmannapeta, Pusuguppa, Tippapuram, Yampuram, and Puttapalli)	Rural and Forested	2012	RDT	1,222	Total: 20.6%^#^ (Maita: 47.6%^#^, Mallampeta: 16.1%^#^, Dharmannapeta: 15.2%^#^, Pusuguppa: 16.9%^#^, Tippapuram: 13.3%^#^, Yampuram: 30.6%^#^, and Puttapalli: 24.5%^#^)	(42)
Cross-sectional	DU/ANC	Madhya Pradesh (Rewa)	Hyperendemic with 62–80% of cases due to *Pv*	2014	LM	203	35.5%^#^	(43)
Cross-sectional	DU	Madhya Pradesh (Katni, Maihar)	Katni (Semi-rural), Maihar (Rural)	2006–2007	Histology/LM/PCR/RDT	506 (histology), 504 (incision smear), 505 (impression smear), 504 (LM), 506 (RDT), and 110 (PCR)	10.3%^‡^ (histology), 4.9%^‡^ (incision smear), 3.6%^‡^ (impression smear), 5.4%^#^ (LM), 4.2%^#^ (RDT), and 34.5%^#^ (PCR)	(44)^**^
Retrospective	ANC	Karnataka (Mangaluru)	-	2014–2015	LM	12,600	0.3%^#^	(45)
Cross-sectional	DU/ANC	Jharkhand (Hazaribag)	Rural and semi-urban district with low but perennial transmission of malaria	Not specified	LM	1,271 (ANC), 870 (DU)	5.4%^#^ (ANC), 4.3%^#^ (DU)	(46)
Cross-sectional	DU/ANC	Chhattisgarh (Bastar, Rajnandgaon)	Rural and Forested (Bastar is high endemic, Rajnandgaon is low endemic)	2007–2008	LM/PCR	2,477 (ANC), 948 (DU)	LM: 1.2%^#^ (ANC) and 1.7%^‡^ (DU); PCR: 3.4%^#^ (ANC) and 4.2%^‡^ (DU)	(47)
Cross-sectional	-	Uttar Pradesh (Aligurgh)	Not specified	Not specified	LM/QBC/RDT	156	57.0%^#^	(48)^$^
Descriptive	ANC	Chhattisgarh, Andhra Pradesh, and Telangana	Forested	2015	RDT	563	29.3%^#^	(49)
Cross-sectional	DU/ANC	Rajasthan (Bikaner)	Seasonal transmission	Not specified	qPCR/LM	ANC: 2,021 (LM) and 298 (qPCR), DU: 1,206 (LM) and 297 (PCR)	LM: 1.3%^#^ (ANC) and 0%^‡^ (DU)	(50)
							qPCR: -^#^(ANC) and -^#^(DU)	
Case–Control	ANC	Jharkhand (Hazaribag)	Rural and semi-urban district with low and perennial transmission of malaria	2014–2015	LM/PCR/RDT	534	9.4%^#a^	(51)
Cross-sectional	ANC	Karnataka (Mangaluru)	-	2014–2017	LM/RDT	105	67.6%^#^	(52)
Cluster randomized controlled trial	ANC/DU	Jharkhand (Kamdara and Basia in Gumla district, Bano and Kolebira in Simgeda district)	Forested with malaria peak from June to October	2012–2015	RDT/PCR/Histology	ANC	ANC	(53)
						ISTp: 3,163 (RDT) and 2,620 (PCR)	ISTp: 3.2%^#^ (RDT) and 5.9%^#^ (PCR)	
						PCD: 108 (RDT) and 2,706 (PCR)	PCD: 9.3%^#^ (RDT) and 4.2%^#^ (PCR)	
						DU	DU	
						ISTp: 1,405 (RDT) and 1,454 (Histology)	ISTp: 2.2%^‡^ (RDT) and 6%^‡^ (Histology)	
						PCD: 1,540 (RDT) and 1,560 (Histology)	PCD: 2.1%^‡^ (RDT) and 4.2%^‡^ (Histology)	
Cross-sectional	-	Chhattisgarh (Durg, Sarguja, Bilaspur, Raipur, and Bastar)	Rural and forested with high malaria burden	2019	RDT	21,572	0.8%^#^ (from 0.03% in Durg to 4.4% in Bastar)	(54)^§^
Case–Control	ANC/DU	Madhya Pradesh (Maihar)	Rural and semi-urban, and ethnic tribal populations	2010–2012	LM	3,873	1.3%^#^ & 1.3%^‡^	(55)
Cross-sectional	-	Karnataka (Mangaluru)	-	2015	LM/RDT	29	20.7%^#^	(56)^§^

ANC, Antenatal care visit; DU, Delivery unit; ISTp, Intermittent screening and treatment during pregnancy; MiP, Malaria in pregnancy; *Pf*, *Plasmodium falciparum*; *Pv*, *Plasmodium vivax*; LM, Light microscopy; PCR, Polymerase chain reaction; PCD, Passive case detection; QBC, Quantitative buffy coat; qPCR, Quantitative PCR; and RDT, Rapid diagnostic test. The list of studies used is presented as Supplementary Table 1. ^†^Characteristics of the area at time of study. ^#^Maternal peripheral blood was used for malaria parasite detection. ^‡^Maternal placental blood was used for malaria parasite detection. ^&^Placental infection included presence of malarial parasite and/or hemozoin (malarial pigment). ^§^The study was conducted in community. ^$^The study included women attending hospital and presenting signs suggestive of severe malaria. ^**^Histology was the gold standard as method for malaria parasite detection. ^a^Only *Pv* infections.

### 3.3. Plasmodium species involved in MiP

**Figure 3:**
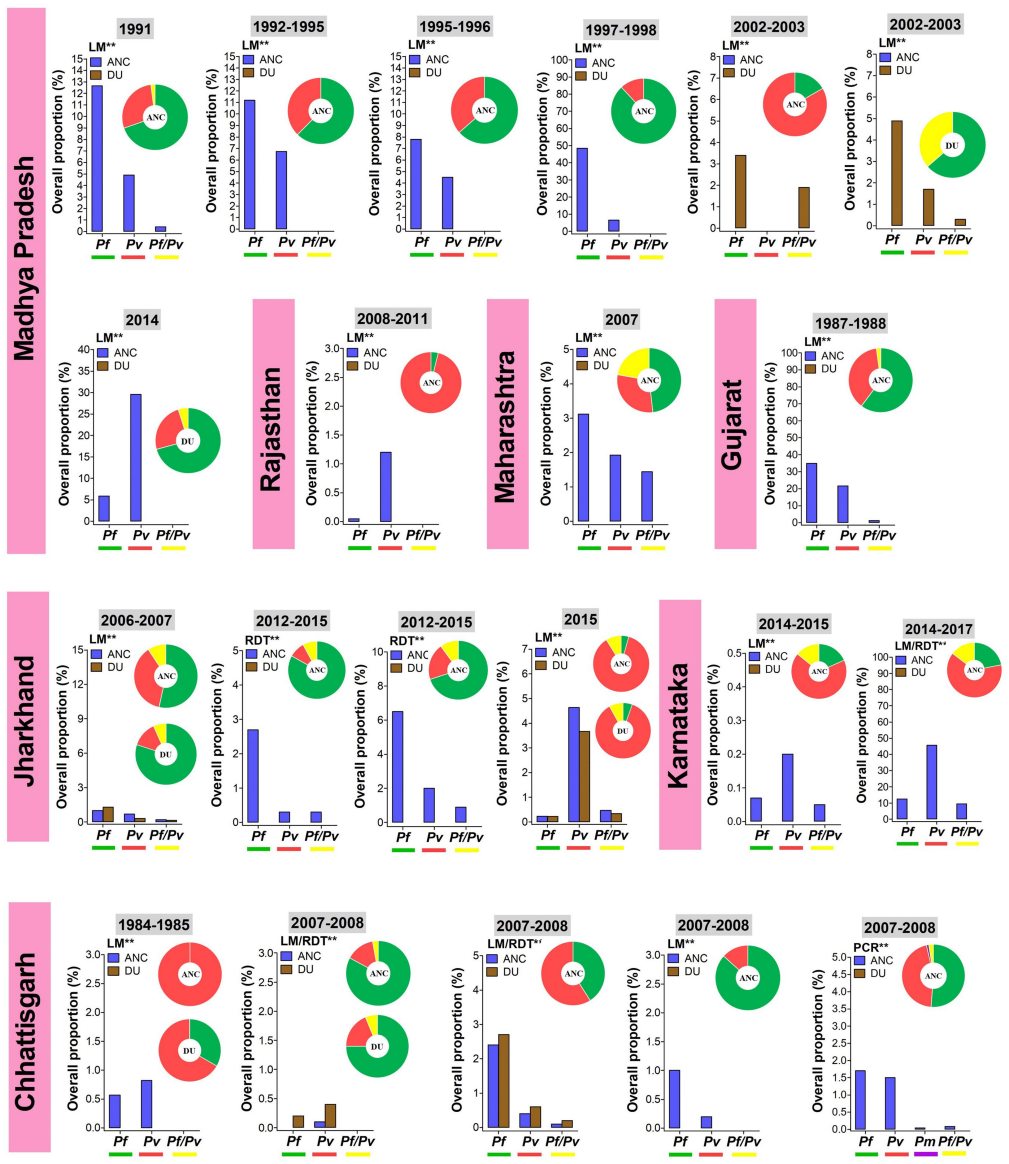
Overall proportion and contribution of *Plasmodium* species either as peripheral mono- or mixed infections in ANC and DU by states. ANC, Antenatal care visit; DU, Delivery unit; LM, Light microscopy; RDT, Rapid diagnostic test; PCR, Polymerase chain reaction; *Pf*, *Plasmodium falciparum*; *Pm*, *Plasmodium malariae*; *Pv*, *Plasmodium vivax*; *Pf/Pv*, Mixed infection with *P. falciparum* and *P. vivax.* Bars represent overall proportions of *Plasmodium* infections which are computed as ratio of number of patients with *Plasmodium* species either mono-infection or mixed infections to total number of patients. Pie charts represent the specific proportion of *P. falciparum*, *P. ovale*, *P. malariae*, and *P. vivax* species either mono- or mixed infections. These proportions were computed as ratio of total number of patients with either one *Plasmodium* species either mono- or mixed infections to total number of *Plasmodium*-infected patients. Findings were stratified by timing of screening (ANC and DU). *Pf* mono-infections, *Pv* mono-infections, and *Pf*/*Pv* mixed infections are depicted in green, red, and yellow, respectively. ^**^Diagnostic method used for detection *Plasmodium* infections.

**Figure 4:**
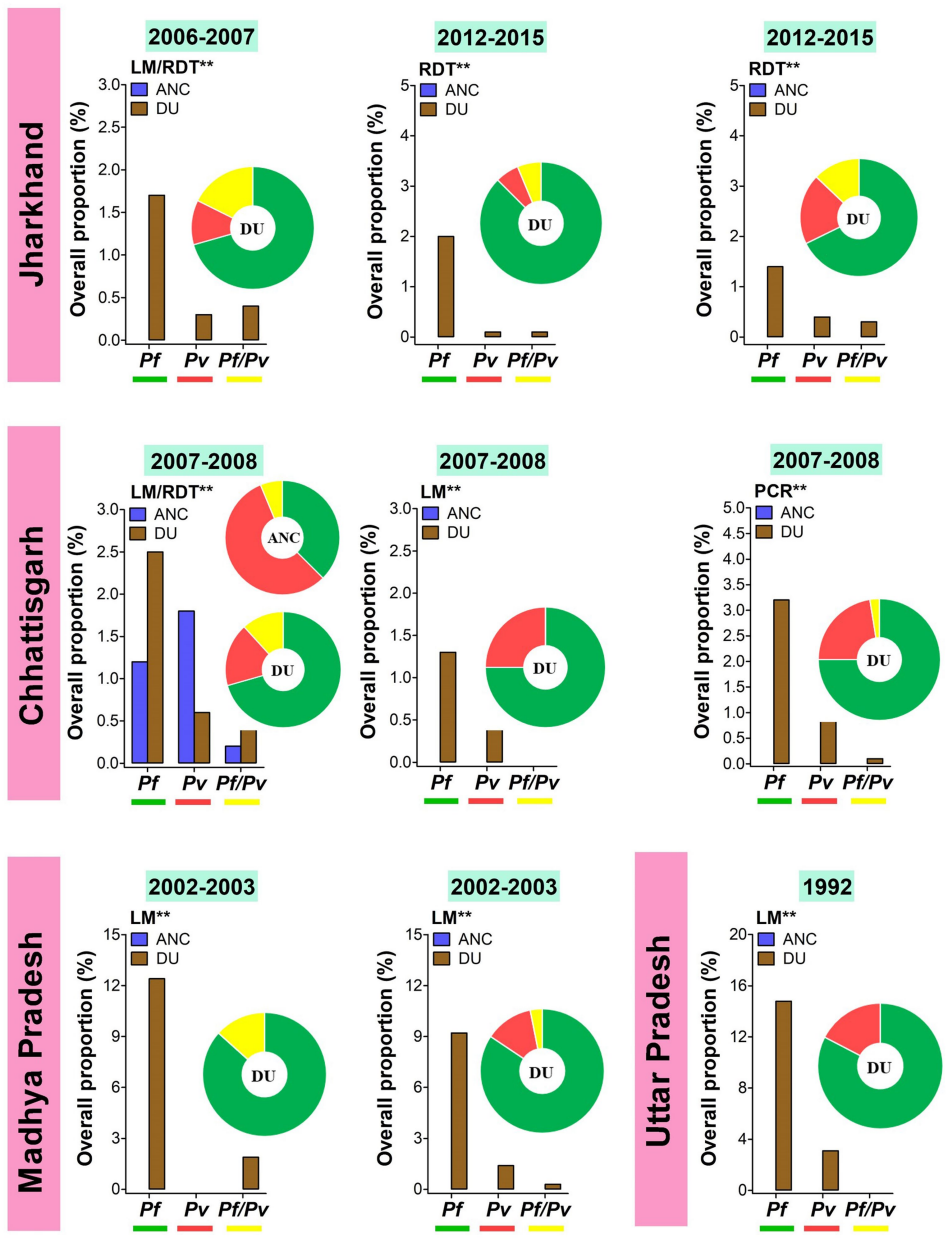
Overall proportion and contribution of *Plasmodium* species either as placental mono- or mixed infections in ANC and DU by states. ANC, Antenatal care visit; DU, Delivery unit; LM, Light microscopy; RDT, Rapid diagnostic test; PCR, Polymerase chain reaction; *Pf*, *Plasmodium falciparum*; *Pv*, *Plasmodium vivax*; *Pf/Pv*, Mixed infection with *P. falciparum* and *P. vivax*. Bars represent overall proportions of *Plasmodium* infections which are computed as ratio of number of patients with *Plasmodium* species either mono-infection or mixed infections to total number of patients. Pie charts represent the specific proportion of *P. falciparum*, *P. ovale*, *P. malariae*, and *P. vivax* species either mono- or mixed infections. These proportions were computed as ratio of total number of patients with either one *Plasmodium* species either mono- or mixed infections to total number of *Plasmodium*-infected patients. Findings were stratified by timing of screening (ANC and DU). *Pf* mono-infections, *Pv* mono-infections, and *Pf*/*Pv* mixed infections are depicted in green, red, and yellow, respectively. ^**^Diagnostic method used for detecting *Plasmodium* infections in the studies.

The information on *Plasmodium* species-wise MiP proportion is mainly originated from hospital based studies, in a limited number of states such as Madhya Pradesh, Jharkhand, Maharashtra, Rajasthan, and Chhattisgarh (Figure 2). *Pf* and *Pv* are predominant species involved in MiP irrespective of diagnostic method and timing of screening. In Madhya Pradesh, *Pf* was the main MiP-associated malaria species on ANC visits and DU with overall peripheral *Pf* mono-infections prevalence of 3.4–48.5% based on LM (Figure 3) (28, 30, 31, 33, 36, 43). Likewise, a trend of *Pf* dominance was also seen in Chhattisgarh with LM-based placental prevalence of *Pf* mono-infections ranging from 1.2 to 3.2% (Figure 4), even though the contribution of plasmodial species can vary within the same state (41, 47, 57). Singh and colleagues conducted a study in two districts of Chhattisgarh with different malarial endemicity level (i.e., Rajnandgaon and Bastar), and showed that *Pv* was dominant in Rajnandgaon (low endemic area) both at ANC and DU while *Pf* was dominant in Bastar (high endemic area; Figure 3) (41). The same team reported *Pm* as additional cause of MiP in Chhattisgarh using PCR method (47). Similarly, *Po* was detected as mixed infection with *Pf* in a multicentric study (58). One study from Rajasthan reported the predominance of *Pv* species which accounted for 96.2% of all LM-detected peripheral infections among women attending ANC visits (Figure 3) (50).

Temporal analysis of burden of MiP was limited in regions such as Madhya Pradesh, Jharkhand, and Chhattisgarh (Figures 3, 4). In Madhya Pradesh, the prevalence of peripheral *Pf* infection at ANC decreased from ~13% in 1991 to ~7% in 1995–1996, while prevalence of peripheral *Pv* infection at ANC levels off at ~5% between 1991 and 1997–1998. In Jharkhand, the prevalence of *Pv* infection at ANC increased from ~1% in 2006–2007 to ~5% in 2015. The same trend was observed for peripheral *Pf* infection among women attending ANCs in Chhattisgarh where the prevalence of this species increased from ~0.5% in 1984–1985 to ~1.5% in 2007–2008 (Figure 3). In contrast, an increase in prevalence of peripheral *Pv* infection at DU was noted in Jharkhand (~0.5% in 2006–2007 to ~4% in 2015). Regarding placental infection, the burden of *Pf* infection slightly decreased from ~1.5% in 2006–2007 to ~1% in 2012–2015 in women living in Jharkhand (Figure 4). There are few studies that analyzed temporal trends of MiP burden in other settings such as sSA and Latin Americas where malaria endemicity patterns are different. One study reported an apparent reduction in *Pf* MiP burden from ~60 to 5% during years 1994–2019 in different malaria eco-epidemiological regions (coastal savannah zone, middle forest zone, and northern savannah zone) in Ghana (59).

### 3.4. Clinical and parasitological features of MiP

Clinical presentation of MiP in India is diverse with *Plasmodium* infections ranging from asymptomatic carriage of the parasites to severe clinical forms.

#### 3.4.1. Asymptomatic malaria

The detection of *Plasmodium* parasitemia of any density, in the absence of fever or other acute symptoms, in individuals who have not received recent antimalarial treatment is known as asymptomatic malaria (60). All plasmodial species can elicit asymptomatic infections during pregnancy with higher parasitemia in *Pf* infections compared to non-*Pf* infections (50). Data on asymptomatic MiP are greatly lacking from India. The burden of asymptomatic malaria varies across Indian areas, and this heterogeneity is related to several factors such as using diagnostic techniques with varying sensitivity and specificity. Using RDT, Corrêa and colleagues reported that ~10% of *Plasmodium spp*-infected women were asymptomatic in tribal areas of Chhattisgarh, Telangana, and Andhra Pradesh (49). In Jharkhand state, it was reported that >70% of MiP cases were asymptomatic in a randomized trial (53). Another study in the same state reported same results both, at ANC (70.6%) and DU (75.7%) (46). Similarly, a community based study found that asymptomatic cases accounted for 76.7% of all malaria cases in rural areas in Chhattisgarh (54). There is a link between asymptomatic infections and parasite density, with many asymptomatic infections being found at submicroscopic levels and thus can only be detected using molecular tools (61). A recent meta-analysis reported overall prevalence of submicroscopic *Plasmodium* infections of 0.4–38.4% in general Indian population (62), which is lower estimates as seen in MiP. In Chhattisgarh, Singh and colleagues found significant proportion of both peripheral and placental *Plasmodium* spp. submiscroscopic infections, for which the extent was species-dependent, among majorly primigravidae/secundigravidae women (47). More than 60% of peripheral and placental infections were submicroscopic with higher rates seen in *Pv* compared to *Pf* infections (placental: 66.7 vs. 64.7%, peripheral: 90.5 vs. 50%) (47). Tracking asymptomatic and submicroscopic *Plasmodium* infections is vital for malaria elimination strategies in endemic areas (63), especially in India where malaria burden significantly decreased in last years.

#### 3.4.2. Uncomplicated malaria

The fraction of asymptomatic infections that will become clinically symptomatic is shaped by a cocktail of factors such as level of transmission, control measure coverage, comorbidities (e.g., human immunodeficiency virus—HIV, malnutrition), host and parasite factors (64, 65). Uncomplicated malaria (UM) encompasses non-pathognomonic and flu-like signs and symptoms which often comprise fever, nausea, rigors, chills, headache, muscle pains, etc. (66). Symptoms usually occur 7–30 days after mosquito bite and last for 6–10 h sequentially three stages: cold, hot, and sweating (67). The same clinical malaria symptomatology is seen in pregnant women living in endemic areas such as India. Most of the *Pf* and *Pv* UM-related symptoms reported in Indian pregnant women attending ANC and/or DU included fever, weakness/fatigue, body/joint pain, headache, loss of appetite, diarrhea, dizziness, hepato-splenomegaly, and nausea/vomiting (33, 43, 44, 46, 49, 50, 52, 54).

Malaria is also associated with cell and biochemical changes in multiple organs and tissues. One of the most dominant MiP-associated signs is anemia for which risk is increased in pregnant women and malaria-infected pregnant women (46, 55). The pathophysiological mechanism includes hemolysis of both *Plasmodium*-infected and uninfected red blood cells (RBCs) and impaired/suppressed hematopoiesis (67, 68). The sequestration pathophysiological phenomenon is known and described well in *Pf* parasites (69), but not for *Pv* and *Pk* though few reports showed ability of *Pv*- and *Pk*-infected RBCs to cytoadhere to endothelial cells in placenta, bloodstream vessels and brain even though low cytoadherence of *Pk*-infected RBCs to cerebral microvascular endothelial cells was found (70–72). In India, malarial anemia during pregnancy is due to *Pf* and *Pv* with overall prevalence of 36.6–100% varying across the country (39–43, 46, 47, 50, 58, 73), with mild and moderate forms accounting for >85% of all anemia cases (28, 41, 46, 49). The extent of MiP-associated anemia seems to be higher in primigravidae/secundigravidae and ANC patients. On ANC visits anemia prevalence of ~67–88.3% against ~59.6–83.9% at DU through studies conducted in Jharkhand, Chhattisgarh, and Rajasthan (39, 46, 47, 50).

#### 3.4.3. Severe malaria

Pregnant women are particularly susceptible to *Plasmodium* infections and its severe forms in malaria endemic regions of India (31, 57, 74). Available data outline that *Pf* is the principal contributor to severe MiP cases and maternal/fetal outcomes, and few reports about *Pv* causing SM attacks (75, 76). No severe MiP case due to non-*Pf/Pv* species has been documented in the world so far. Using systematic review and meta-analysis approach, we recently showed that the overall prevalence of SM in individuals with *Pv* mono-infection was 29.3% in India, with lowest and highest rates in Karnataka (15.3%) and Uttarakhand (57.8%), respectively (5). In pregnant women, data on SM prevalence are greatly missing in India, and the studies on clinical patterns of severe MiP are focused and/or have evaluated few particular presentations only (e.g., ARDS, CM) (77, 78). One study from Karnataka reported that 32.4% of malaria-infected patients were diagnosed with SM, and *Pv* was the main contributor of SM cases (56.6%) (52). Most of severe *Pf* MiP cases occurred in primigravidae as reported by Singh et al. in Madhya Pradesh (29), and Kochar et al. in Rajasthan (79). No severe MiP case with *Po*, *Pm*, and *Pk* have been documented in India till now.

##### 3.4.3.1. Severe malarial anemia

Malaria infection is a risk factor for severe anemia in Indian pregnant women (47, 50). Based on the available data, severe anemia is found at prevalence of 3–15.6% in *Plasmodium*-infected pregnant women (28, 39, 41, 46, 47, 49, 50, 52, 74). *Pf* and *Pv* as mono- and mixed infections are the species responsible for this severe hematological condition, with higher rates seen in *Pf*-MiP as reported in Karnataka state (52). However, these estimates do not reflect the real burden of MiP related SMA in Indian context for at least four reasons: (i) different thresholds for hemoglobin level were used for diagnosing severe anemia (e.g., Hb < 5 g/dL or < 7 g/dL), (ii) in some studies, moderate and severe anemia were collectively diagnosed with the same Hb threshold (e.g., Hb < 9 g/dL) (41), (iii) very few studies appraised other severe anemia-inducing conditions such as malnutrition (49), and (iv) none of the studies included parasitemia threshold for defining SMA as per WHO guidelines (Tables 2, 3). In this context, it is needed to document the real contribution of SMA in pregnancy in India.

**Table 3:** Severe clinical manifestations of MiP in malaria infected individuals in India.

States	Severe anemia	CM	ARDS/PE	Hypoglycemia	Jaundice	Shock	ARF	Prostration	Multiple convulsions	Acidosis	Bleeding	Multiorgan dysfunction	Malaria species^f^	Ref.
Chandigarh	-	7%	-	-	-	-	-	-	-	-	-	-	*Plasmodium*	(57)
Madhya Pradesh	11.5%^c^	-	-	-	-	-	-	-	-	-	-	-	*Plasmodium*	(28)
Rajasthan	20% ^a^	75.5%	4.4%	6.7%	13.3%	-	20%	-	11.1%	-	-	13.3%	*Pf*	(80)
Rajasthan	-	-	13.3%^&^	-	-	-	-	-	-	-	-	-	*Pf*	(81)
Orissa	-	60%	-	-	-	-	-	-	-	-	-	-	*Pf*	(82)
Rajasthan	-	76%	-	-	-	-	-	-	-	-	-	-	*Pf*	(79)
Madhya Pradesh	-	7%	-	-	-	-	-	-	-	-	-	-	*Pf*	(31)
Multiple states^d^	38%	-	3%	17%^#^	7.4%	-	8%^#^	-	13%	-	-	-	*Plasmodium*	(58)
Gujarat	-	-	-	-	-	-	4.2%^‡^	-	-	-	-	-	*Plasmodium*	(83)
Jharkhand	3.9–4.5%^b^	-	-	-	-	-	-	-	-	-	-	-	*Plasmodium*	(39)
Madhya Pradesh	-	-	9.5%^&^	-	-	4.7%	-	-	-	-	-	-	*Plasmodium*	(40)
Rajasthan	60%^a^	0%	4%	4%	12%	-	8%	0%	-	-	-	4%	*Pv*	(74)
Gujarat	-	2%	0%	20%	4%	2%	0%	2%	-	-	0%	0%	*Pv*	(84)
Chhattisgarh	10.7–15.6%^b^	-	-	-	-	-	-	-	-	-	-	-	*Plasmodium*	(47)
Jharkhand	7.8–13.6%^b^	-	-	-	-	-	-	-	-	-	-	-	*Plasmodium*	(46)
Uttar Pradesh	-	26.9%	-	35%	-	-	0%	-	16.8%	-	-	-	*Pf*	(48)
Rajasthan	7.5–8.6% ^b^	-	-	-	-	-	-	-	-	-	-	-	*Pv*	(50)
Multiple states^e^	6.9%^b^	-	-	-	-	-	-	-	-	-	-	-	*Plasmodium*	(49)
West Bengal	-	-	-	29%	6%	-	3%	-	-	-	-	-	*Pf*	(85)
Karnataka	11.3%^a^	-	-	-	-	-	-	-	-	-	-	-	*Plasmodium*	(52)
Jharkhand	0.7–1.1% ^b^	-	-	-	-	-	-	-	-	-	-	-	*Plasmodium*	(53)
Andhra Pradesh	12%^b^	22%	3%	3%	14%	4%	8%	-	12%	4%	5%	-	*Plasmodium*	(86)

ARDS, Acute respiratory distress syndrome; ARF, Acute renal failure; CM, Cerebral malaria; PE, Pulmonary edema; *Pf*, *Plasmodium falciparum*; *Pv*, *Plasmodium vivax*; WHO, World Health Organization; Ref., References. Estimates are percentages of each severe clinical manifestation among malaria-infected pregnant women, unless otherwise indicated. ^#^Only reported in *Pf* parasites. ^&^Pulmonary edema. ^‡^Malaria was responsible for 4.2% of all ARF analyzed in the study. ^a^Hemoglobin < 5 g/dL was used for defining for severe anemia. ^b^Hemoglobin < 7 g/dL was used for defining for severe anemia. ^c^Hemoglobin threshold for defining for severe anemia was not specified. ^d^The study was conducted in nine Indian states (Orissa, Meghalaya, Tripura, Assam, Mizoram, Manipur, Sikkim, Andhra Pradesh, and Chhattisgarh). ^e^The study was conducted in four Indian states (Chhattisgarh, Andhra Pradesh, and Telangana). ^f^The estimates in the studies were found for *Plasmodium* spp., *Pf*, or *Pv*.

##### 3.4.3.2. Cerebral malaria

Cerebral malaria is a common severe manifestation in Indian pregnant women (38, 77, 84, 87, 88), as high CM prevalence of 60 and 76% reported among malaria-infected individuals from Orissa and Rajasthan states, respectively (79, 82). Routinely, CM is more frequently seen in primigravidae/secundigravidae compared to multigravidae, and is mostly associated with poor maternal and fetal outcomes (28, 31, 77, 88).

##### 3.4.3.3. Hypoglycemia

Hypoglycemia is often reported at low rates in Indian children and non-pregnant adults diagnosed with SM. A systematic review and meta-analysis found a pooled proportion of hypoglycemia of 0.05% due to *Pv*-related mono-infections in the country (5). In SM pregnant women, prevalence of hypoglycemia is ~3–35% with higher rates found in *Pf* infected pregnant women compared to their *Pv* infected counterparts (Table 3). In Rajasthan, *Pv* induced hypoglycemia in 4% of malaria patients while a prevalence of 35% was reported among *Pf*-infected women in Uttar Pradesh (Table 3) (48, 74).

##### 3.4.3.4. Acute renal failure

The etiology of acute renal failure (ARF) is multifactorial in pregnancy, and studies outline that malaria contributes toward a small fraction as reported from Gujarat state where ARF proportion is ~0–8% in pregnant women (83). A study from Rajasthan reported a *Pf*-induced ARF proportion of 20%, but this estimate was obtained on only 45 pregnant women (Table 3) (80).

##### 3.4.3.5. Acute respiratory distress syndrome/pulmonary edema

Acute respiratory distress syndrome is diagnosed in malaria infected pregnant women at lower rates compared to SMA, CM, and ARF, with overall proportion of ~0–4% in states including Rajasthan, Gujarat, and Andhra Pradesh (Table 3). ARDS can pose a veritable diagnostic and therapeutic dilemma as previously reported in a *Pv*-infected preeclamptic pregnant woman (78). Kochar et al. (81) reported that pulmonary edema occurred in 13.3% of malaria infected pregnant women just after delivery in Rajasthan.

##### 3.4.3.6. Clinical jaundice

Jaundice is the main clinical manifestation seen in *Pv* SM in Indian patients (5). This condition occurs in malaria infections due to intravascular hemolysis, disseminated intravascular coagulation, and rarely the occurrence of hepatocellular jaundice in *Pf* malaria infection also known as “malarial hepatitis” (89). Compared to severe *Pf* infections, the risk of jaundice is lower in severe *Pv* infections regardless of the living area and age group (5). In *Plasmodium* spp. infected pregnant women, the proportion of clinical jaundice ranges from ~4–14% with similar rates for *Pf* and *Pv* (Table 3).

##### 3.4.3.7. Other severe malaria clinical manifestations

Severe clinical manifestations such as shock, prostration, metabolic acidosis, and abnormal bleeding can also occur in *Plasmodium* infected pregnant women, but at lower rates (≤ 5%) compared to the above mentioned clinical manifestations (Table 3).

#### 3.4.4. Other severe clinical manifestations: severe thrombocytopenia

Severe thrombocytopenia defined as level of blood platelets below 50,000/μL, is not considered as clinical marker of SM in latest WHO guidelines (10). Previous works in India reported significant fraction of SM patients presenting this hematological disorder (5, 90, 91). *Pf* and *Pv* are able to induce severe thrombocytopenia, and available data in India on comparative analysis of these two species outline that the risk of this condition varies with area. In Karnataka, the risk of severe thrombocytopenia is nearly four times higher in *Pf*-infection compared to *Pv*-infections. In contrast, this risk is reduced by ~60% in *Pf*-infections in Rajasthan state (5). In pregnant women, few studies reported disparate findings for severe thrombocytopenia prevalence in Karnataka (6 and 26.8%) for *Plasmodium* spp. infections while 56 and 82% were found in Rajasthan and Gujarat for *Pv* mono-infections, respectively (45, 74, 84, 86).

### 3.5. Comorbidities and concurrent infections seen in MiP

**Figure 5:**
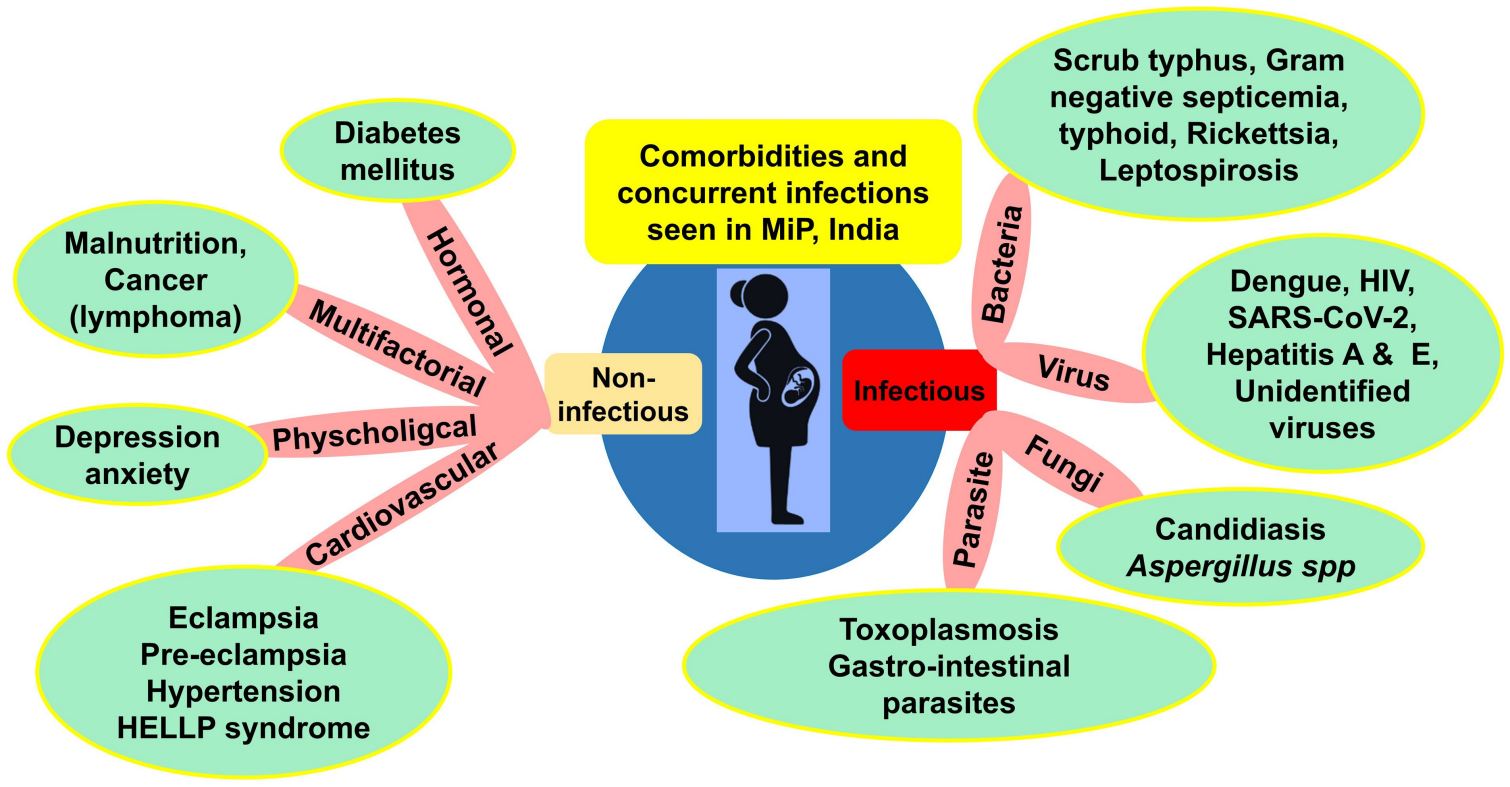
Comorbidities and concurrent infections reported in MiP in India. HELLP, Hemolysis, elevated liver enzymes, low platelet count; HIV, Human immunodeficiency virus infection; MiP, Malaria in pregnancy; SARS-CoV-2, Severe acute respiratory syndrome Coronavirus 2; UTI, Urinary tract infections. Some examples of comorbidities are presented in the green round shapes based on published literature and unpublished reports from health facilities. ^*^Gestational hypertension is included in hypertension cases.

Clinical course and outcomes of MiP are modeled by complex interaction between host, parasite, and environment (48). In addition, external factors such as comorbidities and concurrent infections can modulate interaction between host and *Plasmodium* parasites, and thus impact the natural history of malarial infection in pregnant women (Figure 5).

Concurrent infections included diseases caused by bacteria, viruses, parasites, and fungi. In late 80s, Mehta and Mehta reported toxoplasmosis in *Plasmodium* infected pregnant women from Kolkata, West Bengal (92). In the same period in Chandigarh, septicemia of bacterial origin was reported in one woman diagnosed with CM (57). High diversity of viruses including dengue virus, HIV, and SARS-CoV-2 were concurrently found in malaria infected pregnant women in the country (Figure 5). In Uttar Pradesh, a case report of co-infection with *Pf*, *Pv*, and dengue virus was seen in one 6-month pregnancy (93). In Maharashtra, a co-infection case of *Pv* with SARS-CoV-2 responsible for the current COVID-19 pandemics was described (94).

Regarding comorbidities, mostly cardiovascular disorders such as eclampsia and pre-eclampsia were diagnosed in *Pv*–induced ARDS and CM cases in Delhi and Karnataka were seen (78, 95). The effect of poor nutritional status on malaria risk is still elusive (96). A study from three states (Chhattisgarh, Andhra Pradesh, and Telangana) found malaria infected pregnant women with poor nutritional status (i.e., mid-upper arm circumference < 230 mm) (49). Other comorbidities such as cancer, diabetes and HELLP syndrome have also been reported (Figure 5).

### 3.6. Factors associated with MiP in India

#### 3.6.1. Parity and gestation trimester

**Figure 6:**
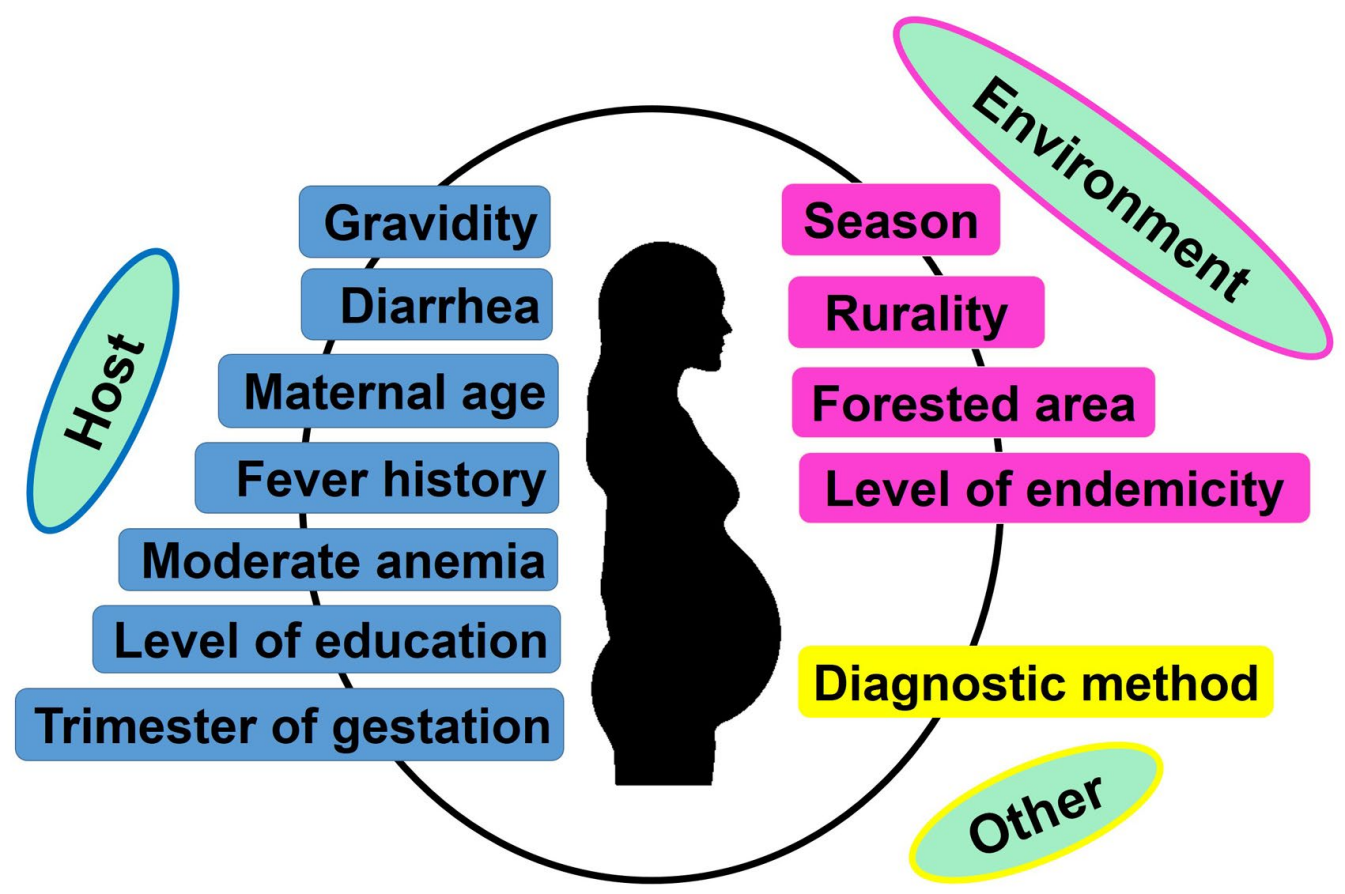
Factors associated with peripheral and/or placental *Plasmodium* spp. infection during pregnancy. Only factors identified based on multivariate logistic regression as reported in each included study were retained to create the image.

**Figure 7:**
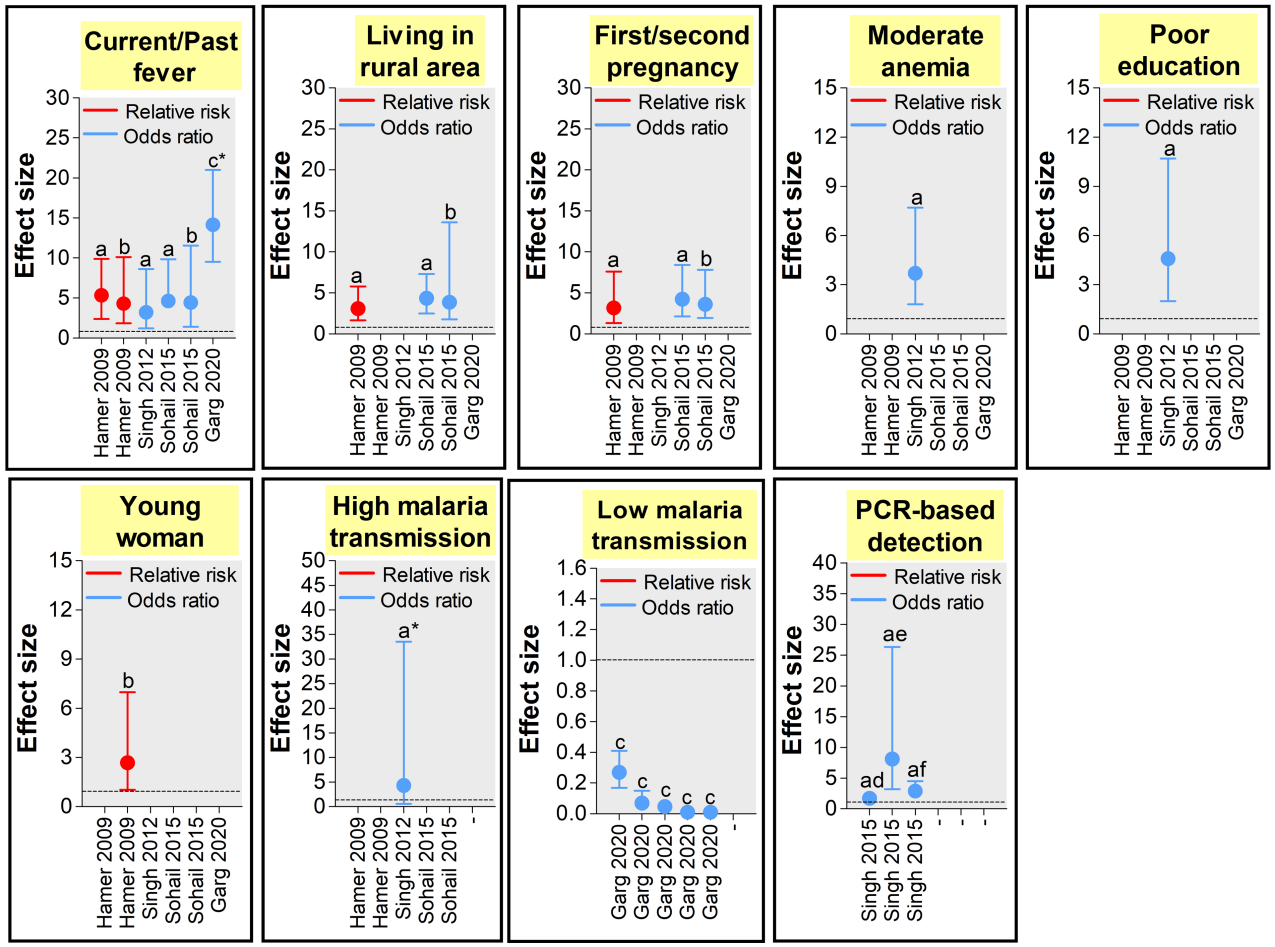
Size effects of peripheral *Plasmodium* spp. MiP determinants in India (39, 41, 46, 47, 54). Effect size was appraised as odds ratio (OR) and relative risk (RR) as reported in each study used to build the graphs. Only study that reported statistically significant effects sizes were retained. Dashed line represents a OR or RR = 1 (neutral factors). Factors with OR/RR < 1 and > 1 represent protective and risk factors of malaria infection, respectively. ^a^Estimated from pregnant women attending antenatal care visits. ^b^Estimated from pregnant women attending delivery units. ^c^Estimated from pregnant women in community. ^d^Estimated for *Plasmodium falciparum* infections. ^e^Estimated for *Plasmodium vivax* infections. ^f^Estimated for *Plasmodium* spp. infections. ^*^The estimates presented in the graph are 1/10 of real estimates found in the study.

Parity is one of the most important factors associated with both peripheral and placental MiP, with primigravidae much at risk of malaria infection and its deleterious consequences (97). Moreover, studies found that the level of antibodies inhibiting placental sequestration of *Pf* parasites is increasing over successive pregnancy, thereby supporting a parity-dependent acquired antimalarial immunity (98, 99). In India, many studies reported both higher peripheral and placental malaria prevalence in primigravidae/secundigravidae compared to multigravidae (27, 29, 31, 32, 39, 46, 73, 79). In Jharkhand, a study reported that peripheral infection risk was 4.23 times higher in primigravidae/secundigravidae compared to multigravidae (46). The same team reported that the risk of placental infection was increased by >3 times in primigravidae/secundigravidae, finding that was previously reported in a study conducted in the same state (Figures 6, 7) (39, 46). It has been reported that *Plasmodium* spp. infection rates are higher during second trimester of pregnancy (65). Data from several states including Madhya Pradesh, Rajasthan, Gujarat also support this observation (27, 31, 33, 80). However, one study conducted in Madhya Pradesh reported highest infection rates during third trimester (30).

#### 3.6.2. Woman’s demographical, clinical and genetic characteristics

The influence of pregnant woman’s characteristics on malaria infection risk has been reported from studies conducted in India, with role of age, level of education, and clinical symptomatology. In Jharkhand, Hamer et al. (39) found that women aged <20 years attending ANC visits had 2.68 times more risk of peripheral *Plasmodium* infection compared to their older counterparts (Figures 6, 7). Similarly, an increased risk for peripheral *Plasmodium* infection was seen in women with lack of formal education in two studies conducted in Chhattisgarh and Jharkhand, respectively (39, 41). Studies reported higher *Plasmodium* infection in febrile pregnant women with signification association between diarrhea, fever or history of fever, moderate anemia and peripheral/placental infection (41, 46, 54). Fever was a stronger determinant of peripheral and placental *Plasmodium* infections both at ANC and DU (41, 46, 54).

#### 3.6.3. Residence area

Several aspects of the residence area have been associated with MiP in India and include level of urbanization, forest cover and level of malaria endemicity (39, 41, 46, 54). In Jharkhand, MiP is more prevalent in rural areas compared to urban areas. Indeed, the risk of peripheral *Plasmodium* infection is ~4–6 times higher in rural women compared to their counterparts from urban areas. Again, the risk of placental *Plasmodium* infection is increased by ~3–4 times in rural areas compared to urban areas in Jharkhand (Figures 6, 7) (39, 46). Working in two areas of Chhattisgarh differing by malaria endemicity, Singh and colleagues reported peripheral *Plasmodium* infection risk increased by ~45 times in pregnant women living in Bastar (high endemicity area) compared to those living in Rajnandgaon (low endemicity area) (41).

#### 3.6.4. Diagnostic methods

Given detection sensitivity of malarial diagnosis tools varies, it is expected to have higher chances of detecting low (very low) *Plasmodium* parasitemia with molecular tools which have higher sensitivity than LM and RDTs (63). Using PCR and LM for detecting peripheral/placental *Pf* and *Pv* infections, Singh et al. pointed out that chances of detecting peripheral/placental infections using PCR were increased by ~2.5–2.9 times for *Plasmodium* spp. infections, ~1.7–2.6 times for *Pf* infections and ~ 2.5–8.1 times for *Pv* infections among women attending ANC and DU in Madhya Pradesh (Figures 6, 7) (47).

### 3.7. Maternal outcomes of MiP

**Figure 8:**
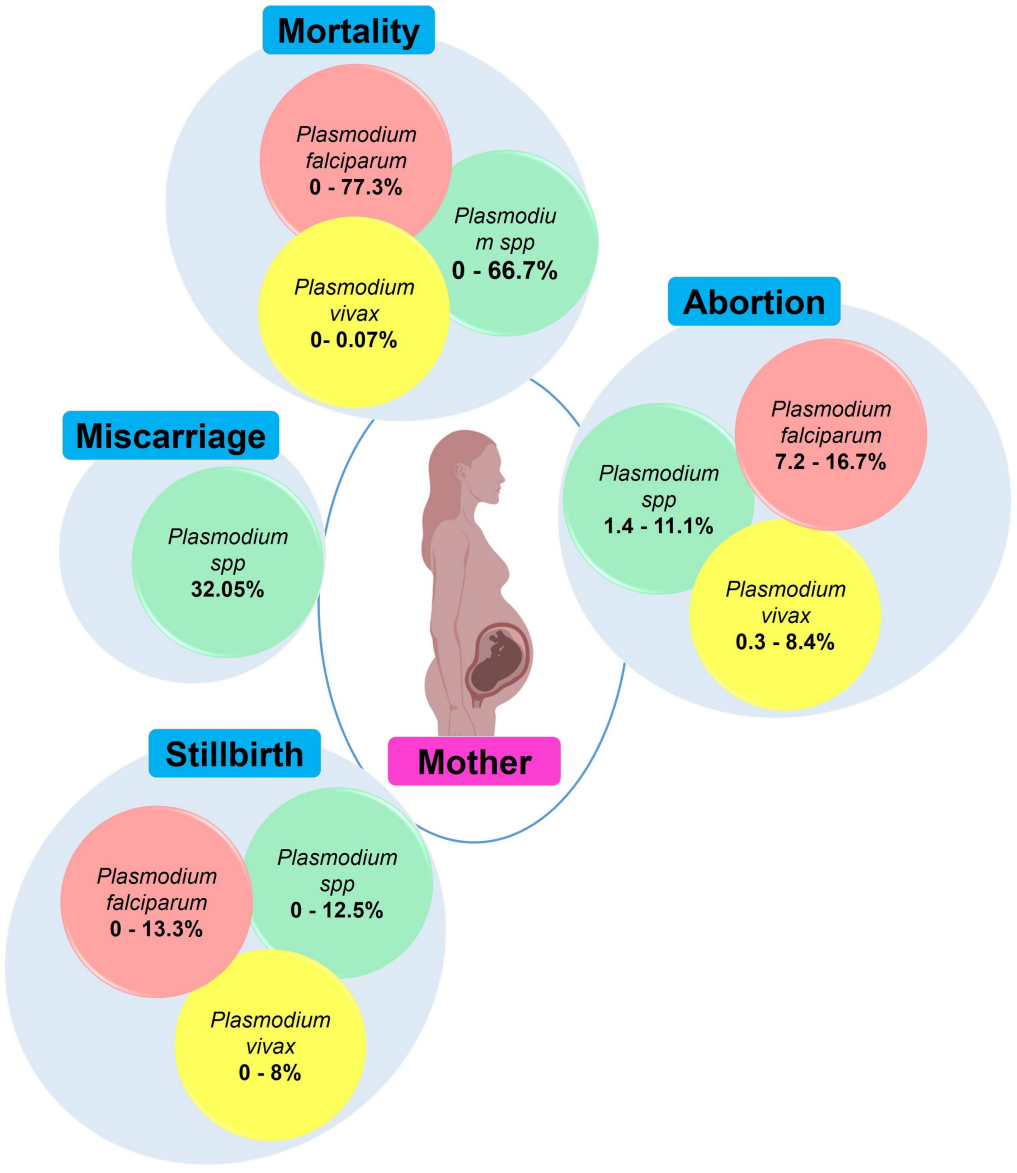
Main maternal outcomes during malaria in pregnancy. The values in round shapes are proportions of each maternal outcome among pregnant women infected with *Plasmodium* spp. (red), *P. falciparum* (red), and *P. vivax* (yellow). The values represent range (minimum and maximum values) for each maternal outcome as reported in each study included in this review. In *Plasmodium* spp., the malarial species was not specified during association analysis with maternal outcomes in the included studies. Created with Biorender.com.

Malaria in pregnancy has a devastating impact on health of mothers and their babies, and is an important cause of maternal and infant mortality in malaria endemic regions. In malaria endemic areas *Plasmodium* infections are associated with adverse maternal outcomes such as miscarriage, stillbirth, abortion, and mortality. In India, *Pf* and *Pv* have been associated with these maternal outcomes which are frequently seen in primigravidae mothers (Figure 8; Supplementary Table 2) (30, 31, 33, 80). CM, pulmonary edema and hypoglycemia were cause of maternal death reported in two studies conducted in Chandigarh and Gujarat (57, 73). The overall prevalence of MiP-related maternal death in malaria-infected individuals is ~0–77.3% in India, with disproportion among different states. *Pf* is the main contributor to maternal death while few rare death cases associated with *Pv* infections have also been reported (50, 100). Abortions and stillbirths are reported at prevalence of ~7.2–16.7 and ~ 0–13.3% for *Pf*, and 0.3–8.4 and ~ 0–8% for *Pv*, respectively. The highest values of *Pf*-related abortions and stillbirths were reported in Rajasthan among pregnant women presenting with SM (80, 81).

Only two studies from Chhattisgarh and Rajasthan quantified the risk of maternal outcomes in MiP (47, 50). Using LM, Singh and colleagues found that pregnant women with peripheral *Plasmodium* spp. infection had 1.8 times higher risk of anemia and 13.7 times higher risk of severe anemia that their uninfected counterparts (Supplementary Table 3). Also, the risk of LBW was nearly six times higher in women with placental *Plasmodium* spp. infections compared to that with no infection (47). In Rajasthan, clinical *Pv* infection was associated with five-higher risk of maternal anemia (50). Likewise, the risk of maternal anemia was increased by four times in women with microscopic *Pf* infection compared to uninfected women. In the same vein, women with placental microscopic *Pf* infections were 4.28 times more at risk to give birth babies with LBW compared to those with no infection (50) (Supplementary Table 3).

### 3.8. Fetal/neonatal/infancy outcomes of MiP in India

**Figure 9:**
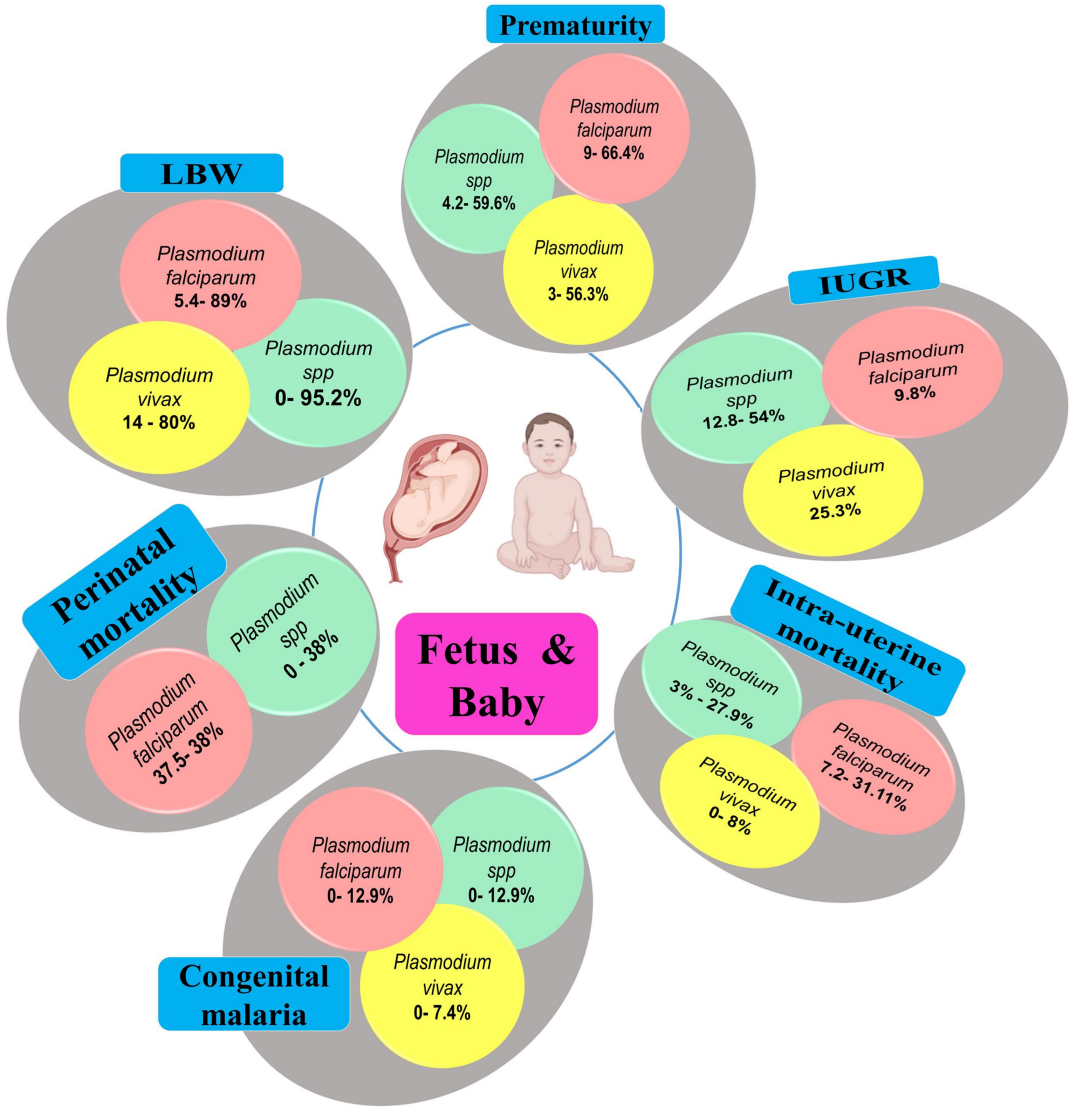
Main fetal/neonatal outcomes during MiP, India. IUGR, Intra-uterine growth retardation; LBW, Low birthweight; MiP, Malaria in pregnancy. The values in round shapes are proportions of each fetal/neonatal outcome among babies infected with *Plasmodium* spp. (red), *P. falciparum* (red), and *P. vivax* (yellow). The values represent range (minimum and maximum values) for each fetal/neonatal outcome as reported in each study included in this review. In *Plasmodium* spp., the malarial species was not specified during association analysis with fetal/neonatal outcomes in the included studies. Created with Biorender.com.

Placental malaria infections are associated with adverse outcomes on fetus, newborns, and even during infancy. Malaria parasites, especially *Pf*, have a high tropism for placenta tissue in which the parasites develop and collaterally induce important histological changes (e.g., fibrinoid necrosis, calcification) (44). Such *Plasmodium*-induced placental changes impair fetal-maternal exchange and lead to disastrous consequences in babies such as low birth weight (LBW), prematurity, intrauterine growth retardation (IUGR), respiratory distress, and deaths in the worst case (Figure 9; Supplementary Table 3). LBW, IUGR and prematurity are the predominant adverse outcomes in India, with *Pf* and *Pv* as main contributors.

Studies outlines that ~5.4–89 and ~ 14–80% of *Pf*- and *Pv*-infected women gave birth babies with LBW, respectively. In Madhya Pradesh, it was reported that almost all babies (95.2%), born to malaria infected women, had LBW (33, 36). Similarly, the prevalence of prematurity can often surpass 50% for both *Pf* and *Pv* in Indian women. It was reported that severe maternal anemia was risk factor for both LBW and prematurity in Jharkhand (101). Growth restricted babies are also frequently seen during MiP with prevalence of ~12.8–54, ~0–9.8, and ~ 0–25.3% for *Plasmodium* spp.*, Pf*, and *Pv*, respectively (Figure 9; Supplementary Table 4). It is now recognized that *Pv* can also cause poor birth outcomes including perinatal and intrauterine mortality, but at lower extent than its *Pf* counterpart. In Indian context, the prevalence of *Pv*-induced intrauterine death during MiP ranges from 0 to 8% while that of *Pf* is estimated at ~7.2–31.1% (Figure 9; Supplementary Table 4).

Malaria in pregnancy and its birth consequences are consistently correlated with increased malaria risk during infancy (102, 103). A study found an increased 2-fold risk for malaria infection and clinical malaria in small-for-gestational age babies born to Beninese women (104). Such data are scarce in India, but in one study conducted in Madhya Pradesh, mothers and their infants were followed up for 1 year. The authors reported increase in malaria prevalence, intensity and frequency during the follow up, and three of all *Pf*-infected infants died before their first birthday (33).

### 3.9. Neonatal and congenital malaria

In clinical practice, congenital malaria is defined as presence of *Plasmodium* asexual stages in cord blood and peripheral blood of the baby during first week of life (105). In neonatal malaria, *Plasmodium* asexual stages are found in neonates aged ≤28 days (105). A recent meta-analysis estimated global NCM prevalence at 40.4 and 12%, due to several variable factors including area and detection methods (106).

**Figure 10:**
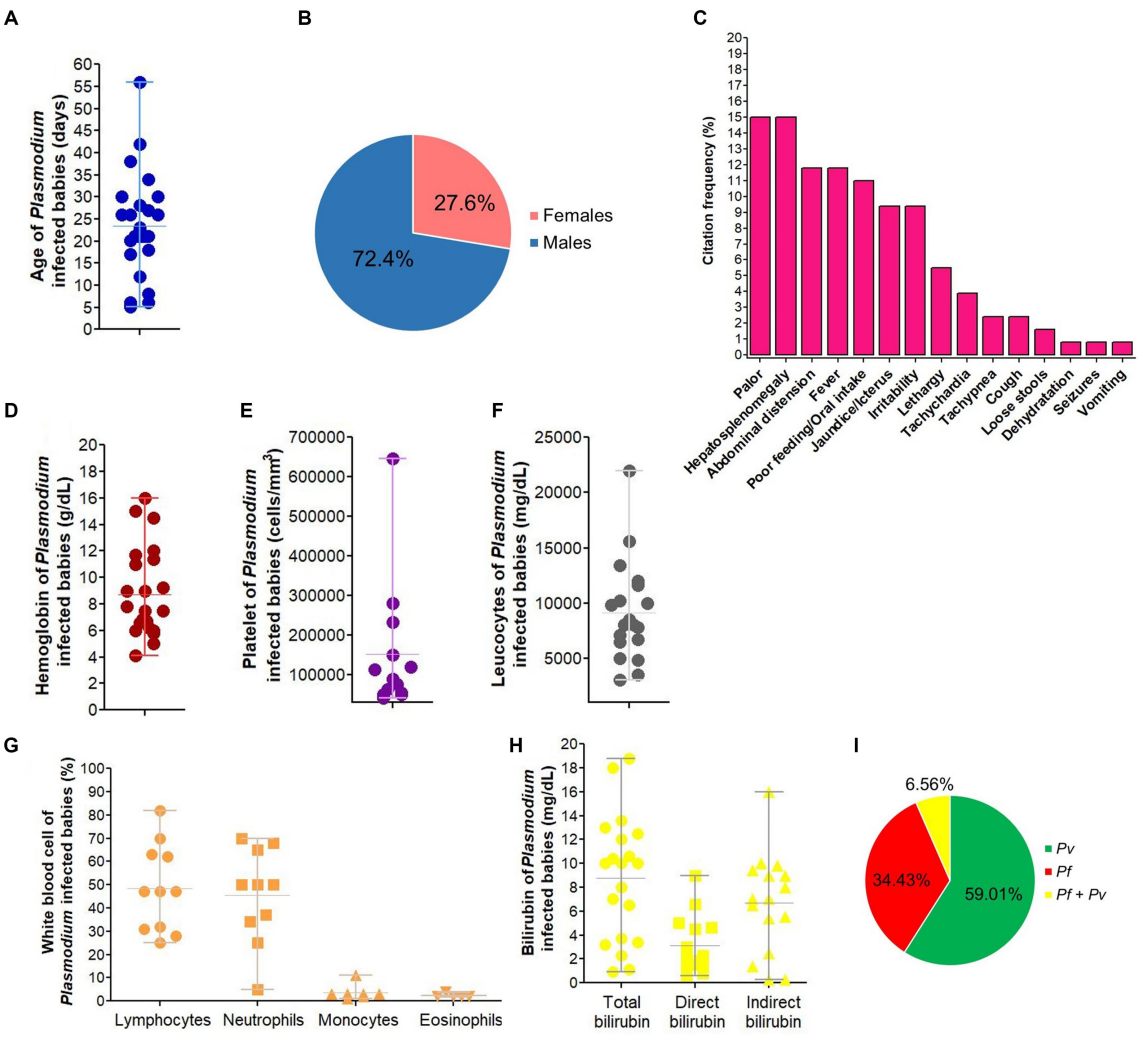
Characteristics of neonatal malaria cases in India. *Pf*, *Plasmodium falciparum*, *Pv*, *Plasmodium vivax*. The image depicts characteristics of *Plasmodium* infected babies for age **(A)**, gender-distribution **(B)**, signs/symptoms presented on admission/birth **(C)**, hemoglobin level **(D)**, platelet count **(E)**, total leucocytes **(F)**, white blood cell populations **(G)**, total, direct and indirect bilirubin level **(H)**, and malaria infection **(I)**. We excluded data from congenital malaria due to very low number of cases (*n* = 3). In panel **(C)**, percentage of each symptom on admission were computed by dividing the number of times that symptom was cited to the total number of citations for all symptoms. In panel **(I)**, malaria species were identified using LM and/or RDT.

The prevalence of congenital malaria in India ranges from 0 to 12.9% (Figure 9). Prevalence data on neonatal malaria are absent in India, but some case reports outlined its occurrence in the country (Supplementary Table 5). NCM cases have been reported throughout India especially in states such as West Bengal, Madhya Pradesh, Rajasthan and Uttar Pradesh (Supplementary Tables 3–5). In India, NCM cases are mostly born to primigravidae women, have LBW and are aged 26 days on average with male predominance. On admission, babies present at hospital with mosaic of signs/symptoms mostly including pallor, hepatosplenomegaly, fever, jaundice/icterus, and irritability. Anemia, thrombocytopenia, leucopenia and clinical jaundice are frequently seen in newborns infected with malaria parasites having parasitemia in ranges from 0.1 to 25% and *Pv* mono-infections account for ~60% of all LM/RDT-detected NCM cases (Figure 10; Supplementary Table 5).

### 3.10. Prevention of MiP

In last 2 decades, India greatly scaled up large number of malaria prevention methods throughout the territory to achieve elimination objectives by 2030 (107, 108). Malaria prevention in India relies essentially on free provision of ITNs/LLINs and IRS (109). LLINs are recommended in high malaria risk areas even though no formal system exist for their distribution during ANC visits (110). Complementary control strategies such as chemoprophylaxis, sensitization campaigns for behavior changes, reduction of breeding sites for mosquito vectors and early case detection and prompt treatment are also encouraged by the Government of India. Ownership and use rates of LLINs vary greatly within and between states, and even when LLINs are present in household they are not adequately used by Indian population (111, 112). A large scale household based study reported LLINs use rates of 89 and 91% among pregnant women in the Odisha state (113). Similar rate (88.6%) was reported in pregnant women in another study conducted in the same state (114). In contrast, available data from health facility-based studies suggest that ITNs/LLINs use rates are lower than those seen in community studies. The proportion of pregnant women using ITNs/LLINs most of nights is ~18.3–82.8% and that of women sleeping under ITNs/LLINs the last night is ~0.05–82.5% (39, 41, 44, 47, 50, 52, 115). Also, the utilization of untreated ITNs/LLINs by pregnant women was common in few areas (39, 46). IRS is generally less used in households by pregnant women compared to ITNs/LLINs, with use rates of ~0–58.5% (39, 41, 44, 47, 50). Taking malaria chemoprophylaxis is very uncommon in pregnant women as per the studies conducted in forested and tribal areas of Jharkhand, Chhattisgarh, Rajasthan, and Madhya Pradesh (39, 41, 44, 46, 47, 50).

### 3.11. Therapeutic approaches for MiP control and drug resistance in India

Prevention of MiP with IPTp-SP is not implemented in India, and control of the disease in pregnancy relies on passive case detection during ANC visits. Until 2010, the Indian national guidelines recommended the utilization of quinine for *Pf* malaria and CQ for *Pv* malaria regardless the trimester of gestation (116). Since then, this MiP treatment policy changed with regard to Indian state, malaria species, severity of the infection and trimester of gestation. For uncomplicated *Pv* infections, CQ is administered in all trimesters. The utilization of primaquine (PQ) for radical cure of *Pv* infection is not recommended to pregnant women and her fetus. The treatment of uncomplicated *Pf* MiP is CQ for first trimester women while ACTs artemether + lumefantrine (AL) or artesunate + sulfadoxine/pyrimethamine (AS + SP) are used for second and third trimesters. Due to high level of SP resistance in Northeastern states, AL is recommended for treatment while AS + SP is used in other states. Parenteral quinine, artesunate, or artemether are given for SM (6, 116, 117).

Drug efficacy studies are limited in MiP in the country, but existing data outline both high efficacy and safety of AS + SP and AS + MEF (118). CQ and SP resistance are both well established, and studies reported low frequency *Pfk13* mutations associated with artemisinin resistance (i.e., 446I, 539 T, and 561H) in Arunachal Pradesh and West Bengal (119–121). Several mutations in the *Pfk13* gene—i.e., F446I, N458Y, C469Y, M476I, Y493H, R539T, I543T, P553L, R561H, P574L, C580Y, R622I, and A675V—are strongly associated with resistance of *Pf* parasites to ACTs, the current antimalarial drugs recommended for treatment of uncomplicated *Pf* malaria (1, 119). There is a dearth of data on antimalarial drug resistance status in MiP. Using *in vitro* and *in vivo* studies, some authors found that 100 and 31.4% of *Pf* isolates collected from Madhya Pradesh and Uttar Pradesh states were resistant to CQ (34, 48). Although not purposely designed for appraising drug resistance, other studies reported adequate clinical and parasitological response of ~19.7–79% in *Pf*-infected pregnant women treated with CQ at health facilities (28, 30, 58).

### 3.12. Challenges and future directions on MiP in India

**Figure 11:**
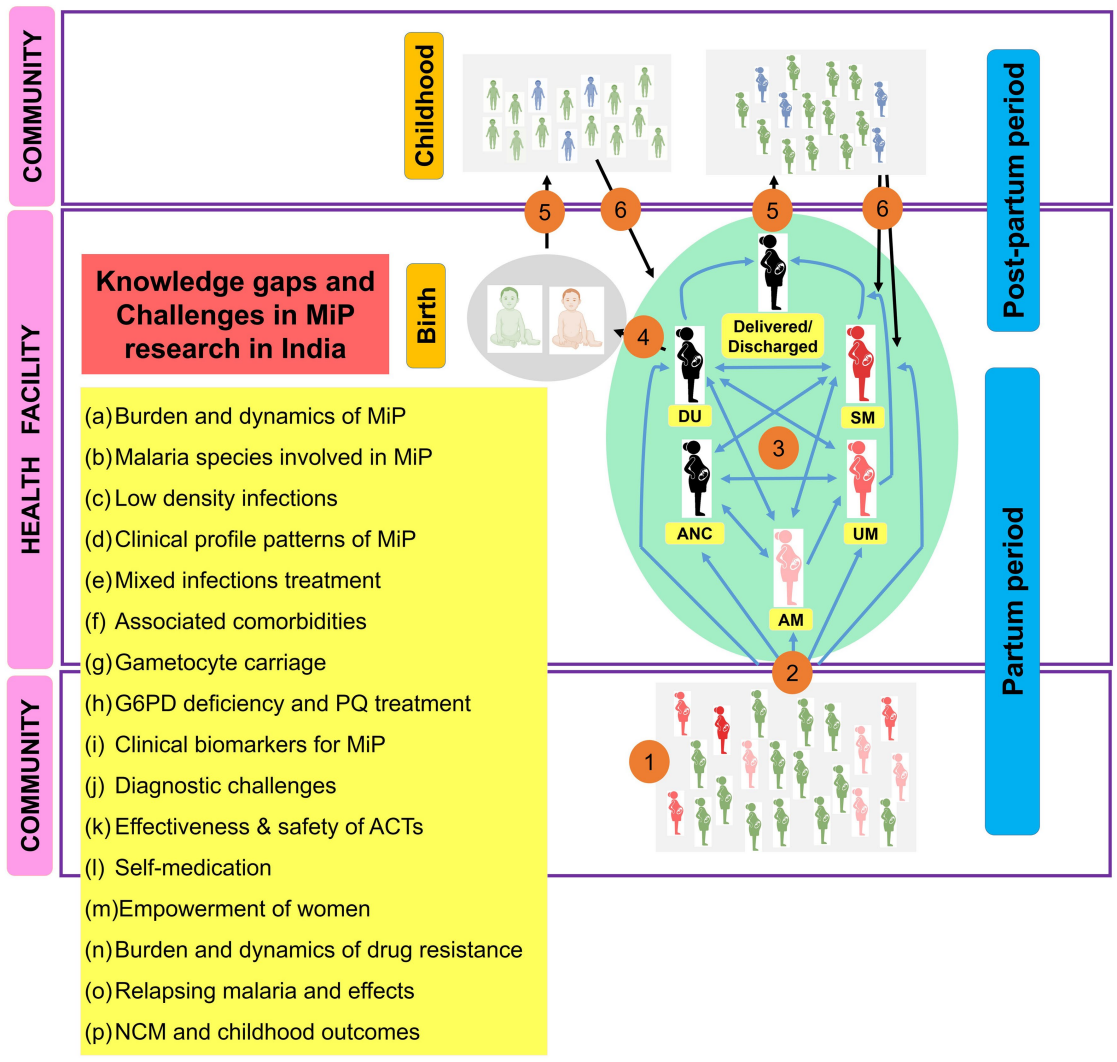
Main research gaps and challenges to be addressed on MiP in India. ACTs, Artemisinin based combination therapies; AM, Asymptomatic malaria; ANC, Antenatal care; DU, Delivery unit; G6PD, Glucos-6-phosphate dehydrogenase; MiP, Malaria in pregnancy; NCM, Neonatal and congenital malaria; PQ, Primaquine; UM, Uncomplicated malaria; SM, Severe malaria. The image presents the main knowledge gaps and challenges on MiP research in India. (1) At community, pregnant women are exposed to infecting *Anopheles* bites and some of them will be infected (red). The plasmodial infection may exist as asymptomatic and/or symptomatic. (2) Both asymptomatic and symptomatic (UM and SM), women can attend health facilities for diverse reasons (e.g., ANC, DU, and care seeking). (3) Women attending ANC/DU can present symptoms or not, and will be managed as per national health guidelines if malaria infection is diagnosed. (4) Women came for delivery can give birth to uninfected babies (gray) or babies diagnosed with congenital malaria (red), and these will be treated as per national guidelines. (5) Women and babies discharged after successful antimalarial treatment will go back home. (6) Women and babies are treated with CQ if *Pv* infection was diagnosed, but using PQ is prohibited for them. Thus, some of them (blue) can develop recurrent malaria episodes during post-partum period and childhood due to reactivation of hypnozoites. During this itinerary for women and babies, several challenges and knowledge gaps (yellow box) have been identified and should be taken into account in future researches in India.

In this quest for achieving malaria elimination objectives, MiP should also be taken into account and a certain number of challenges ranging from prevention to treatment should be investigated in future by Indian researchers. Missing links on MiP research and proposed solutions are presented below and summarized in Figure 11.

Indian researches on epidemiology of MiP have carried out in few states such as Madhya Pradesh and Chhattisgarh, and there is a dearth of cross-sectional/longitudinal studies from other areas including Northeastern states (e.g., Mizoram, Meghalaya, and Tripura), Bihar and Uttarakhand where malaria endemicity is still high. Also, more studies on MiP and NCM in tribal and forested settings are also greatly needed (122, 123).

Minor species including *Po* and *Pm* are also associated with MiP in India (22). LM is the standard and reports pinpointed that *Po* and *Pm* infections are misdiagnosed as *Pv* and *Pf* infections using LM in the country (124, 125). It is worth determining the extent and characterizing these species in MiP as previous studies showed their ability to elicit SM and noted pathophysiological differences between *P. ovale curtisi* and *P. ovale wallikeri* (4, 126–129).

In general, MiP studies are conducted in health facility settings either during ANC or DU periods. It would be helpful to more document MiP in community setting especially the extent of asymptomatic and/or submicroscopic parasitemia and their impact on clinical course of MiP. In addition, non-*Pf* low density infections (LDI) especially *Pv* parasitemia are problematic given efficient transmission of *Pv* LDI to *Anopheles* vectors (130, 131).

Clinical presentation of MiP in India encompasses asymptomatic, uncomplicated and severe malaria. In the latter clinical form, SMA is predominantly seen during pregnancy in India. However, other factors such as malnutrition and helminthiasis may also cause SMA, but these are rarely concomitantly addressed in MiP studies. Macronutrient undernutrition is a big contributor to severe adverse outcomes during pregnancy (132), but such studies are lacking in India.

As above discussed national guidelines for treatment of MiP are different for *Pf* and *Pv* species. However, studies report high rates of mixed infections on field that can hinder control of malaria in India (133, 134). Also, treatment of malaria in presence of comorbidities such as HIV may also be tricky and impact pregnancy outcome (e.g., drug–drug interactions). It was reported that severity and mortality are increased in patients co-infected with *Plasmodium* parasites and HIV (135, 136).

Pregnant women are also reservoir for gametocytes, the transmission development stage of *Plasmodium* parasites. Singh and coworkers reported gametocyte carriage rates of 54 and 73.8% in *Pf*-infected pregnant women from Madhya Pradesh (28, 30). This research area is understudied in MiP and should be investigated in future.

*Plasmodium vivax* parasites produce dormant stages called hypnozoites and responsible for malaria relapses, which are associated with transmittable gametocytaemia and delayed mortality (137, 138). PQ is currently recommended for preventing *Pv* relapses but its utilization is associated with risk of severe hemolysis in persons diagnosed with glucose-6-phosphate dehydrogenase (G6PD) deficiency (139). In India, G6PD testing is rarely performed at health facilities coupled with poor adherence to PQ-based 14-day regimen treatment and high prevalence of G6PD deficiency (e.g., Odisha) (5, 140, 141). This limits researches on evaluation of real burden of *Pv* relapses, effectiveness, and development of new hypnozoiticidal drugs.

Immunity against *Pv* is more rapidly acquired than that against *Pf*, that results in high proportion of *Pv* asymptomatic infections (142–144), which are often associated with high carriage of hypnozoites and undetected by LM and RDT (142, 144–146). Development of point-of-care tests based on biomarkers could be promising approach. We recently proposed a simple theoretical framework for identifying, evaluating and validating diagnostic, therapeutic prognostic and predictive biomarkers for malaria, and these could be translated to MiP (147). Longley et al. (148) proposed an interesting approach based on serological markers to detect recent *Pv* infection.

Another cause of lower sensitivity of RDTs in pregnant women could be likely deletions in the histidine rich protein 2 gene (*pfhrp2*) which encodes a *Pf*-specific protein antigen (149). No studies have evaluated *pfhrp2* deletions in pregnant women in India (150, 151), and it would be interesting to appraise the prevalence profile of such deletions at ANC, DU, and community settings.

No studies on SP and artemisinin resistance in MiP in India have been carried out. Also, data on incidence of CQ resistance in MiP, which are probably may parallel CQ resistance in normal *Pf* infections occurring in the same region, are still needed. It is required to conduct more studies especially longitudinal studies to detect temporal variation of the drug resistance gene profile in pregnancy and its association with maternal outcomes.

Even though ACTs are recommended by National guidelines for treating MiP, there is a lack of studies on their effectiveness and safety in India (118). Systematic reviews and meta-analyses of prospective studies and clinical trials conducted in African and Asian settings have showed the efficacy and safety of ACTs during MiP (152, 153). In this context, further investigations are required to determine pharmacological aspects, effectiveness and inocuity profile of ACTs in MiP all around the country.

Self-medication both with traditional medicines and antimalarial drugs should also be addressed in pregnancy. Few studies reported pregnant woman were self-medicating, and this expose them to adverse effects of drugs, often of poor quality as reported in several endemic sSA and SEA areas including India (154, 155).

Some studies reported poor awareness of pregnant women toward malaria, its treatment and prevention due to several causes including social barriers. Thus, there is need to empower pregnant and childbearing women toward malaria and preventive methods and this could be achieved through sensitization during community campaigns and ANC visits.

## 4. Conclusion

Malaria in pregnancy is still a serious public health concern in India. Its epidemiological burden is high in Indian pregnant women, with *Pv* and *Pf* as main causative agents while minor species (*Pm*, *Po*) are also involved. The epidemiology of these species in MiP is greatly varied with important role of mother’s demographic and obstetrical characteristics, geography, and ecoclimatic features of the area. *Plasmodium* infections in pregnant women, often associated with comorbidities and concurrent infections, may progress from asymptomatic carriage of parasites to SM, which is mostly represented by SMA, CM, and hypoglycemia, and more frequently seen in *Pf* infections. MiP has deleterious effects on mother and her child that can often end with death. This review provided a comprehensive overview on epidemiological situation and identified important missing links in MiP and NCM to inform population, clinicians, and researchers. There is urgent need for further studies on the different above mentioned points addressed in the present review. If adequately addressed, the future findings could be greatly helpful for efficiently controlling MiP in India through development, implementation, and scale up of control strategies and policy makers, and thus achieve malaria control and elimination objectives in the country.

## Author contributions

LPKF and VS conceptualized the paper. LPKF conducted literature review and extracted and analyzed data from papers, conceived the figures and maps, performed extraction and analysis data, and drafted the first version of the final manuscript. VS revised the manuscript for important intellectual content and supervised the work at all stages. All authors contributed to the article and approved the submitted version.

## Figures and Tables

**Table:** 

ACT	Artemisinin-based combination therapy
AL	Artemether + Lumefantrine
AM	Asymptomatic malaria
API	Annual parasite incidence
ARDS	Acute respiratory distress syndrome
ARF	Acute renal failure
AS	Artesunate
CM	Cerebral malaria
COVID-19	Coronavirus disease 2019
CQ	Chloroquine
DIC	Disseminated intravascular coagulation
G6PD	Glucose-6-phosphate dehydrogenase
HELLP	Hemolysis, elevated liver enzymes, low platelet count
HIV	Human immunodeficiency virus
IFA	Indirect fluorescence antibody
IL	Interleukins
IPTp	Intermittent preventive treatment during pregnancy
IRS	Indoor residual spraying
ISTp	Intermittent screening and treatment during pregnancy
ITN	Insecticide-treated net
IUGR	Intra-uterine growth retardation
LBW	Low birthweight
LDI	Low density infection
LLIN	Long lasting insecticide-treated net
LM	Light microscopy
MEF	Mefloquine
MiP	Malaria in pregnancy
n.a	Not applicable
NCM	Neonatal and congenital malaria
NVBDCP	National Vector Borne Disease Control Program
OR	Odds ratio
PCR	Polymerase chain reaction
*Pf*	*Plasmodium falciparum*
*Pk*	*Plasmodium knowlesi*
*Pm*	*Plasmodium* malariae
*Po*	*Plasmodium* ovale
*Pv*	*Plasmodium vivax*
PCD	Passive case detection
*pfhrp2*	*Plasmodium falciparum* histidine rich protein 2 gene
QBC	Quantitative buffy coat
RBC	Red blood cell
RDT	Rapid diagnostic test
RR	Relative risk
SARS-CoV-2	Severe acute respiratory syndrome Coronavirus 2
SEA	South East Asia
SM	Severe malaria
SMA	Severe malarial anemia
SP	Sulfadoxine + Pyrimethamine
sSA	sub-Saharan Africa
TNF	Tumor necrosis factor
UM	Uncomplicated malaria
WHO	World Health Organization
