# Supplementary material for: Malaria in pregnancy in India: a 50-year bird’s eye
Source: Front Public Health. 2023 Oct 19;11:1150466. doi: 10.3389/fpubh.2023.1150466 (PMC10620810; doi:10.3389/fpubh.2023.1150466)
Supplement: Supplementary file 1 [file Data_Sheet_1.docx]

Supplementary Material

**Supplemental Table 1**. List of studies on MiP retrieved from the national and international sources

| **Authors** | **Title** | **Topic** | **Design** | **Setting** | **Period** | **Area (s)** |
| --- | --- | --- | --- | --- | --- | --- |
| Sholapurkar et al. (1988a) | Clinical course of malaria in pregnancy - A prospective controlled study from India | Clinical spectrum & Outcomes | Case-Control | Health facility | DU | Chandigarh and neighbouring villages |
| Sholapurkar et al. (1988b) | Malarial parasite density in infected pregnant women from northern India | Outcomes | Case-Control | Health facility | ANC | Chandigarh and neighbouring villages |
| Arya et al. (1989) | Cerebral malaria in pregnancy | Outcomes | Cross-sectional | Health facility | DU | Not specified |
| Prasad et al. (1990) | Malaria infection during pregnancy | Epidemiology | Cross-sectional | Health facility | ANC | Uttar Pradesh |
| Mathur et al. (1991) | Cerebral malaria in pregnancy | Outcomes | Cross-sectional | Health facility | - | - |
| Kaushik et al. (1992) | Malarial placental infection and low birth weight babies | Epidemiology & Outcomes | Cross-sectional | Health facility | DU | Uttar Pradesh (Jhansi) |
| Nair and Nair (1993) | Effects of malaria infection on pregnancy | Outcomes | Cross-sectional | Health facility | DU/ANC | Gujarat (Surat) |
| Maitra et al. (1993) | Maternal manifestations of malaria in pregnancy: a review | Epidemiology & Outcomes | Cross-sectional | Health facility | DU/ANC | Gujarat (Surat) |
| Saikia et al. (1994) | Malaria in pregnancy. A clinical study in an industrial hospital | Outcomes | Cross-sectional | Health facility | ANC | Assam (Digboi) |
| Singh et al. (1995) | Prevalence of malaria among pregnant and non-pregnant women of District Jabalpur, Madhya Pradesh | Epidemiology, Outcomes & Treatment | Cross-sectional | Health facility | ANC | Madhya Pradesh (Jabalpur) |
| Singh et al. (1996) | Malaria parasite density in pregnant women of district Jabalpur, Madhya Pradesh | Epidemiology & Outcomes | Cross-sectional | Health facility | DU/ANC | Madhya Pradesh (Jabalpur) |
| Kochar et al. (1997) | Severe and complicated malaria in Bikaner (Rajasthan), Western India | Outcomes | Cross-sectional | Health facility | DU | Rajasthan (Bikaner) |
| Singh et al. (1998) | Studies on malaria during pregnancy in a tribal area of central India (Madhya Pradesh) | Epidemiology, Outcomes & Treatment | Cross-sectional | Community | - | Madhya Pradesh (Mandla District) |
| Kochar et al. (1998) | Falciparum malaria and pregnancy | Outcomes | Cross-sectional | Health facility | DU/ANC | Rajasthan (Bikaner) |
| Mishra et al (1998) | Complicated falciparum malaria during pregnancy | Outcomes | Cross-sectional | Health facility | ANC | Orissa (Rourkela) |
| Singh et al. (1999) | Epidemiology of malaria in pregnancy in central India | Epidemiology & Outcomes | Cross-sectional | Health facility | DU/ANC | Madhya Pradesh (Jabalpur) |
| Kochar et al. (1999) | Mortality trends in falciparum malaria--effect of gender difference and pregnancy | Outcomes | Cross-sectional | Health facility | DU/ANC | Rajasthan (Bikaner) |
| Das (2000) | Malaria during pregnancy and its effects on foetus in a tribal area of Koraput District, Orissa | Epidemiology & Outcomes | Cross-sectional | Health facility | DU/ANC | Orissa (Koraput) |
| Singh et al. (2001a) | Malaria during pregnancy and infancy, in an area of intense malaria transmission in central India | Epidemiology, Outcomes & Prevention | Cross-sectional | Health facility | ANC | Madhya Pradesh (Mandla District) |
| Singh et al. (2001b) | Status of chloroquine efficacy against Plasmodium falciparum in pregnant women in a tribal area of central India | Epidemiology | Efficacy trial | Community | - | Madhya Pradesh (Mandla District) |
| Singh et al. (2003) | Placental Plasmodium vivax infection and congenital malaria in central India | Epidemiology & Outcomes | Cross-sectional | Health facility | DU | Madhya Pradesh (Mandla District) |
| Konar (2004) | Observations on malaria in pregnancy | Epidemiology, Outcomes & Treatment | Cross-sectional | Health facility |  | National (Orissa, Meghalaya, Tripura, Assam, Mizoram, Manipur, Sikkim, Andhra Pradesh, Chhattisgarh) |
| Singh et al. (2005) | Evaluation of a Rapid Diagnostic Test for Assessing the Burden of Malaria at Delivery in India | Epidemiology | Cross-sectional | Health facility | DU | Madhya Pradesh (Mandla & Satna District) |
| Singh et al. (2005) | Evaluation of a rapid diagnostic test for assessing the burden of malaria at delivery in India | Diagnsotic | Cross-sectional | Health facility | DU | Madhya Pradesh (Mandla & Maihar Districts) |
| Ahmed et al. (2007) | Rapid assessment of the burden of malaria in pregnancy in Madhya Pradesh, India | Epidemiology & Outcomes | Cross-sectional | Health facility | DU/ANC | Madhya Pradesh |
| Chawla and Manu (2007) | Malaria in pregnancy | Epidemiology & Outcomes | Cross-sectional | Health facility | ANC | Maharashtra (Mumbai) |
| Goplani et al. (2008) | Pregnancy-related acute renal failure: A single-center experience | Outcomes | Cross-sectional | Health facility | ANC | Gujarat |
| Diamond-Smith et al. (2009) | Estimating the burden of malaria in pregnancy: a case study from  rural Madhya Pradesh, India | Modelling | Modelling | Health facility | ANC | Madhya Pradesh |
| Hamer et al. (2009) | Burden of malaria in pregnancy in Jharkhand State, India | Epidemiology, Outcomes & Prevention | Cross-sectional | Health facility | DU/ANC | Jharkhand (Ranchi, Konbir, and Gumla) |
| Recker et al. (2009) | Assessing the burden of pregnancy-associated malaria under changing transmission settings | Modelling | Modelling | - | - | Punjab and Haryana |
| Nayak et al. (2009) | Spectrum of vivax malaria in pregnancy and its outcome: a hospitalbased study | Clinical spectrum & Outcomes | Cross-sectional | Health facility | ANC | Rajasthan (Bikaner) |
| Guin et al. (2012) | Placental Malaria Prevalence of Infestation Amongst Febrile Pregnant Women in Central India: Maternal and Perinatal Outcome | Epidemiology, Outcomes & Prevention | Cross-sectional | Health facility | DU/ANC | Madhya Pradesh (Jabalpur) |
| Seal et al. (2010) | Malaria in pregnancy | Outcomes | Case-Control | Health facility | DU/ANC | Kolkata (West Bengal) |
| Singh et al. (2012) | Malaria prevalence among pregnant women in two districts with differing endemicity in Chhattisgarh, India | Epidemiology, Outcomes & Prevention | Cross-sectional | Health facility | DU/ANC | Chhattisgarh (Bastar,Rajnandgaon) |
| Bhadade et al. (2012) | Maternal outcomes in critically ill obstetrics patients: A unique challenge | Outcomes | Cross-sectional | Health facility | DU | Maharashtra (Mumbai) |
| Chauhan et al. (2012) | Maternal Mortality among Tribal Women at a Tertiary Level of Care in Bastar, Chhattisgarh | Outcomes | Cross-sectional | Health facility | DU | Chhattisgargh (Bastar) |
| Qureshi et al. (2014) | Prevalence of malaria and anemia among pregnant women residing in malaria-endemic forest villages in India | Epidemiology & Outcomes | Cross-sectional | Community | ANC | Maita, Mallampeta, Dharmannapeta, Ppusuguppa, Tippapuram, Yampuram, Puttapalli |
| Singh et al. (2014) | Placental and Neonatal Outcome in Maternal Malaria | Epidemiology & Outcomes | Cross-sectional | Health facility | DU/ANC | Madhya Pradesh (Rewa) |
| Ahmed et al. (2014) | Placental infections with histologically confirmed Plasmodium falciparum are associated with adverse birth outcomes in India: a cross-sectional study | Epidemiology & Outcomes | Cross-sectional | Health facility | DU | Madhya Pradesh (Katni, Maihar) |
| Sohail et al. (2015) | Prevalence of malaria infection and risk factors associated with anaemia among pregnant women in semiurban community of Hazaribag, Jharkhand, India | Epidemiology & Risk factors | Cross-sectional | Health facility | ANC | Jharkhand (Hazaribag) |
| Singh et al. (2015) | What is the burden of submicroscopic malaria in pregnancy in central India? | Epidemiology, Outcomes & Prevention | Cross-sectional | Health facility | DU/ANC | Chhattisgarh (Bastar,Rajnandgaon) |
| Jaju and Bhavi (2015) | Convulsions: eclampsia or Malaria or Both! | Comorbidities | Case report | Health facility | DU | Karnataka (Bijapur) |
| Hirani et al. (2015) | Study of clinical profile of P. vivax malaria in pregnancy | Clinical spectrum & Outcomes | Cross-sectional | Health facility | ANC | Gujarat |
| Manzoor Khan et al. (2016) | Prevalence of chloroquine resistant Plasmodium falciparum malaria in pregnant females attending North Indian Hospital | Epidemiology | Cross-sectional | Health facility | Attending | Uttar Pradesh (Aligurgh) |
| Bardaji et al. (2017) | Burden and impact of Plasmodium vivax in pregnancy: A multi-centre prospective observational study | Epidemiology, Outcomes & Prevention | Cross-sectional | Health facility | ANC | Rajasthan (Bikaner) |
| Corrêa et al. (2017) | High burden of malaria and anemia among tribal pregnant women in a chronic conflict corridor in India | Epidemiology & Outcomes | Descriptive | Health facility | ANC | Chhattisgarh, Andhra  Pradesh and Telangana |
| Sharma et al. (2017) | Plasmodium vivax Induced Acute Respiratory Distress Syndrome – A Diagnostic and Therapeutic Dilemma in Preeclampsia | Symptomatology | Case report | Health facility | ANC | Delhi |
| Datta et al. (2017) | Comparative study on antenatal and perinatal outcome of Vivax and Falciparum malaria in a tertiary care hospital of Kolkata, India | Outcomes | Cross-sectional | Health facility |  | West Bengal (Kolkata) |
| Singh et al (2018) | Role of IL-1β, IL-6 and TNF-α cytokines and TNF-α promoter variability in Plasmodium vivax infection during pregnancy in endemic population of Jharkhand, India | Epidemiology & Risk factors | Case-Control | Health facility | ANC | Jharkhand (Hazaribag) |
| Kuepfer et al. (2019) | Effectiveness of intermittent screening and treatment for the control of malaria in pregnancy: a cluster randomised trial in India | Epidemiology | Controlled randomized trial | Health facility | DU/ANC | Jharkhand |
| Chandrashekar et al. (2019) | Malarial anemia among pregnant women in the south-western coastal city of Mangaluru in India | Clinical spectrum & Outcomes | Cross-sectional | Health facility | ANC | Karnataka (Mangaluru) |
| Palem and Pal (2019) | Maternal and fetal outcome of malaria in pregnancy | Epidemiology, clinical sepctrum and outcomes | Retrospective | Health facility | ANC/DU | Karnataka (Mangaluru) |
| Garg et al. (2020) | Malaria prevalence in symptomatic and asymptomatic pregnant women in a high malaria-burden state in India | Epidemiology | Cross-sectional | Community | - | Chhattisgarh (Bastar, Sarguja, Bilaspur, Raipur, Durg) |
| Singh et al. (2020) | Association of Angiopoietin Dysregulation in Placental Malaria with Adverse Birth Outcomes | Biomarker | Case-Control | Health facility | DU/ANC | Madhya Pradesh (Maihar) |
| Rao et al. (2021) | Comparative study of Plasmodium falciparum and Plasmodium vivax malaria in pregnant and non pregnant women in Visakhapatnam, Andhra Pradesh, India | Clinical spectrum & Outcomes | Cross-sectional | Health facility | ANC | Andhra Pradesh (Visakhapatnam) |
| Chandrashekar et al. (2021) | Epidemiology of malaria during pregnancy in Mangaluru city in the southwestern coastal region of India | Epidemiology | Cross-sectional | Community | - | Karnataka (Mangaluru) |

**Supplemental Table 2**. Details of studies on proportion of malaria-associated maternal consequences in Indian pregnant women

| **Studies** | **Area (Place)** | **Species** | **Maternal deaths** | **Abortions** | **Miscarriage** | **Stillbirth** |
| --- | --- | --- | --- | --- | --- | --- |
| Sholapurkar et al. (1988a) | Chandigarh and neighbouring villages | *Plasmodium* | 4% | 1.20% | - | - |
| Maitra et al. (1993) | Gujarat (Surat) | *Pf* | 8.4% (15 cases) | - | - | - |
| Nair and Nair (1993) | Gujarat (Surat) | *Plasmodium* | 3.8% (7.1% Pf & 0% Pv) | 9.7% (10.7% Pf & 8.4% Pv) | - | 5.70% (10.7% Pf & 0% Pv) |
| Saikia et al. (1994) | Assam (Digboi) | *Plasmodium* | 66.60% | 5.30% | - | 7.20% |
| Singh et al. (1995) | Madhya Pradesh (Jabalpur) | *Plasmodium* | 11% | 3.40% | - | 9.60% |
| Singh et al. (1996) | Madhya Pradesh (Jabalpur) | *Pf* | 17 cases (77.3%) | - | - | - |
| Kochar et al. (1997) | Rajasthan (Bikaner) | *Pf* | 33.30% | 16.70% | - | 13.30% |
| Singh et al. (1998) | Madhya Pradesh (Mandla District) | *Plasmodium* | - | 1 case (primigravid) 1.04% | - | 3 cases (primigravid) for Pf, and 1 case (primigravid) for Pv |
| Kochar et al. (1998) | Rajasthan (Bikaner) | *Pf* | - | 11.11% (12.5% in primmi & 10.34% in multi) | - | 13.33% (18.75% in primmi & 10.34% in multi) |
| Mishra et al (1998) | Orissa (Rourkela) | *Pf* | 31% | 7.20% | - | - |
| Singh et al. (1999) | Madhya Pradesh (Jabalpur) | *Plasmodium* | 3 cases/1.22% (Pf only, 2 primigravida + 1 multi), 0% for Pv | 3 cases/1.04% (Pf only, primigravida), 1 case/0.34% (Pv only primi) | - | 2 cases (Pf only, 1 primigravida + 1 multi), 1 case (Pv only primi) |
| Kochar et al. (1999) | Rajasthan (Bikaner) | *Pf* | 38%/17 cases | 11% | - | 13% |
| Das (2000) | Orissa (Koraput) | *Plasmodium* | - | 4.20% | - | 12.50% |
| Singh et al. (2001a) | Madhya Pradesh (Mandla District) | *Plasmodium* | - | 6 cases/3.97% (Pf only, 1 primi + 4 secundigravidae), 1 case (Pv only secundi) | - | 5 cases (Pf only, 2 primigravida + 1 secun + 2 multi) & 0% Pv |
| Konar (2004) | National (Orissa, Meghalaya, Tripura, Assam, Mizoram, Manipur, Sikkim, Andhra Pradesh, Chhattisgarh) | *Plasmodium* | 8.40% | - | 32.05% | - |
| Chawla and Manu (2007) | Maharashtra (Mumbai) | *Plasmodium* | 0% | 11.10% | - | 3.70% |
| Hamer et al. (2009) | Jharkhand (Ranchi, Konbir, and Gumla) | *Plasmodium* | - | - | - | 11.8% (Positive), 4.1% (negative) |
| Nayak et al. (2009) | Rajasthan (Bikaner) | *Pv* | - | 8% | - | 8% |
| Gita et al. (2010) | Madhya Pradesh (Jabalpur) | *Pf* | 18.8% (Peripheral), 28.6% (Placental), 2.5% (Negative) | - | - | 6.25% (Peripheral), 4.76% (Placental), 0.4% (Negative) |
| Seal et al. (2010) | Kolkata (West Bengal) | *Plasmodium* | 12 cases (30%) | yes | - | - |
| Guin et al. (2012) | Madhya Pradesh (Jabalpur) | *Plasmodium* | 28.60% | - | - | - |
| Singh et al. (2012) | Chhattisgarh (Bastar,Rajnandgaon) | *Plasmodium* | - | - | - | Rajnandgaon (placental malaria):0 % (positive) vs 2.5% (negative); Bastar (Placental malaria): 6.3% (positive) vs 2.2% (negative) |
| Ahmed et al. (2014) | Madhya Pradesh (Katni, Maihar) | *Pf* | - | - | - | 4.35% |
| Singh et al. (2014) | Madhya Pradesh (Rewa) | *Plasmodium* | - | - | - | 4.2% (Positive), 3% (negative) |
| Datta et al. (2017) | West Bengal (Kolkata) | *Plasmodium* | 0% for Pf, 0% for Pv |  |  |  |
| Kuepfer et al. (2019) | Jharkhand | *Plasmodium* | - | - | - | 3.1% to 3.3% |
| Palem and Pal (2019) | Karnataka (Mangaluru) | *Plasmodium* | - | - | - | 7.3% to 9.7% |
| Singh et al. (2020) | Madhya Pradesh (Maihar) | *Plasmodium* | - | - | - | 24.30% |
| Rao et al. (2021) | Andhra Pradesh (Visakhapatnam) | *Plasmodium* | 10% | 5% | - | 3% |

**Supplemental Table 3**. Association between MiP, maternal and fetal outcomes

|  |  |  |  |  | **Maternal and fetal outcomes** | | |
| --- | --- | --- | --- | --- | --- | --- | --- |
| **References** | **Blood** | **Species** | **Variables** | **Categories** | **Maternal anemia** | **Severe anemia** | **LBW** |
| Singh et al., 2015 | Peripheral | *Plasmodium spp* | LM infections | Negative | 1 | 1 |  |
|  |  |  |  | Positive | 1.8 (1.0 - 3.3) | 13.7 (5.3 - 32.5) |  |
|  |  |  | PCR based infections | Negative |  | 1 |  |
|  |  |  |  | Positive |  | 3.7 (1.6 - 7.8) |  |
|  | Placental | *Plasmodium spp* | LM infections | Negative |  | 1 | 1 |
|  |  |  |  | Positive |  | 10.6 (2.3 - 38.7) | 5.8 (1.4 - 33.7) |
|  |  |  | PCR based infections | Negative |  | 1 |  |
|  |  |  |  | Positive |  | 5.3 (1.5 - 15.9) |  |
| Bardaji et al., 2017 | Peripheral | *P. falciparum* | PCR based infections | Negative | 1 |  |  |
|  |  |  |  | Asymptomatic malaria | 4.01 (1.59 - 10.11) |  |  |
|  |  |  |  | Clinical malaria | 5.57 (1.22 - 25.34) |  |  |
|  | Placental | *P. falciparum* | PCR based infections | Negative | 1 |  | 1 |
|  |  |  |  | Microscopic infection | 4.07 (1.93 - 8.58) |  | 4.28 (1.75 - 10.44) |
|  |  |  |  | Negative |  |  | 1 |
|  |  |  |  | Asymptomatic malaria |  |  | 4.52 (1.63 - 12.49) |
|  |  |  |  | Clinical malaria |  |  | 4.29 (0.70 - 26.24) |
|  |  | *P. vivax* | PCR based infections | Negative | 1 |  |  |
|  |  |  |  | Asymptomatic malaria | 0.96 (0.37 - 2.44) |  |  |
|  |  |  |  | Clinical malaria | 5.48 (1.83 - 16.41) |  |  |

LBW: Low birthweight, LM: Light microscopy, PCR: Polymerase chain reaction

Statistics presented in the table included odd ratios and their confidence interval at 95%

Only statistically significant results are presented

Level of statistical significance was set at *p* < 0.05 in the included studies

**Supplemental Table 4**. Details of studies on proportion of malaria-associated fetal/neonatal consequences in India

| **Authors** | **Area (s)** | **Species** | **Prematurity** | **APGAR - 5 min** | **Perinatal mortality** | **Intrauterine growth retardation** | **Intrauterine mortality** | **Low birthweight** |
| --- | --- | --- | --- | --- | --- | --- | --- | --- |
| Sholapurkar et al. (1988) | Chandigarh and neighbouring villages | *Plasmodium* | 7 (9%) 4 in primigravidae + 3 multigravidae | - | 3 (4%) 0 in primigravidae + 3 multigravidae | - | 4 (5%) 2 in primigravidae + 2 multigravidae | - |
| Kaushik et al. (1992) | Uttar Pradesh (Jhansi) | *Plasmodium* | - | - | - | - | - | 83%, 2763 Kg (uninfected) vs 2146 (infected) - statistically significant |
| Nair and Nair (1993) | Gujarat (Surat) | *Plasmodium* | 59.60% (62.6% Pf & 56.2% Pv) | - | - | - | 2% | 82% |
| Maitra et al. (1993) | Gujarat (Surat) | *Pf* | - | - | - | - | 31.08% | - |
| Saikia et al. (1994) | Assam (Digboi) | *Plasmodium* | 11% | - | - | - | - | - |
| Singh et al. (1995) | Madhya Pradesh (Jabalpur) | *Plasmodium* | - | - | 0.70% | - | - | - |
| Singh et al. (1998) | Madhya Pradesh (Mandla District) | *Plasmodium* | - | - | - | - | - | - |
| Kochar et al. (1998) | Rajasthan (Bikaner) | *Pf* | 20% (18.75% primi & 20.68% multi) | - | - | - | 31.11% (43.75% primi & 24.13% multi) | - |
| Mishra et al (1998) | Orissa (Rourkela) | *Pf* | 9% | - | - | - | 7.20% | 5.40% |
| Singh et al. (1999) | Madhya Pradesh (Jabalpur) | *Pf* | - | - | - | - | - | 89% (Positive), 38% (negative) |
| Kochar et al. (1999) | Rajasthan (Bikaner) | *Pf* | 20% | - | - | - | 31% | - |
| Das (2000) | Orissa (Koraput) | *Plasmodium* | 4.20% | - | - | - | 27.90% | 25% |
| Singh et al. (2001) | Madhya Pradesh (Mandla District) | *Plasmodium* | - | - | 4 cases/2% (3 born to multigravidae,women infected with Pf, 1 in apparently uninfected) | - | - | 95.2% (infected women) vs 6% (uninfected) |
| Singh et al. (2003) | Madhya Pradesh (Mandla District) | *Pv* | - | - | - | - | - | - |
| Konar (2004) | National (Orissa, Meghalaya, Tripura, Assam, Mizoram, Manipur, Sikkim, Andhra Pradesh, Chhattisgarh) | *Plasmodium* | 47.44% | - | - | 12.82% | 7.69% | - |
| Singh et al. (2005) | Madhya Pradesh (Mandla & Satna District) | *Plasmodium* | - | - | - | - | - | 2.25 Kg (infected) vs 2.40 (non-infected), 95.2% vs 68% (significant) |
| Chawla and Manu (2007) | Maharashtra (Mumbai) | *Plasmodium* | - | - | - | - | 3.70% | 14.80% |
| Hamer et al. (2009) | Jharkhand (Ranchi, Konbir, and Gumla) | *Plasmodium* | 13.3% (Positive), 5.6% (negative) | - | - | - | - | 26.7% (Positive), 20.9% (negative) |
| Nayak et al. (2009) | Rajasthan (Bikaner) | *Pv* | 32% | - | - | 8% | - | 80% |
| Guin et al. (2012) | Madhya Pradesh (Jabalpur) | *Pf* | 25% (Peripheral), 66.4% (Placental), 7.84% (Negative) | 37.5% (Peripheral), 14.3% (Placental), 4.2% (Negative) | 37.5% (Peripheral), 38.0% (Placental), 7% (Negative) | - | - | - |
| Singh et al. (2012) | Chhattisgarh (Bastar,Rajnandgaon) | *Plasmodium* | - | - | - | - | - | Rajnandgaon (placental malaria):20 % (positive) vs 20.8% (negative); Bastar (Placental malaria): 60.0% (positive) vs 29.3% (negative) |
| Singh et al. (2014) | Madhya Pradesh (Rewa) | *Plasmodium* | 6.9% (Positive), 8.4% (negative) | - | 2.8% (Positive), 3% (negative) | - | - | - |
| Ahmed et al. (2014) | Madhya Pradesh (Katni, Maihar) | *Pf* | 20% | - | - | - | - | 37.10% |
| Hirani et al. (2015) | Gujarat | *Plasmodium* | 10% | - | - | - | - | 10% |
| Nagaraj et al. (2015) | Rajasthan (Bikaner) | *Plasmodium* | - | - | - | - | - | - |
| Bardaji et al. (2017) | Rajasthan (Bikaner) | *Pv* | 23% (132/576) | - | - | - | - | 14% (176/1250) |
| Datta et al. (2017) | West Bengal (Kolkata) | *Plasmodium* | 50% | more than 70% had APGAR below 7 | 4.70% | - | - | more than 50% |
| Kuepfer et al. (2019) | Jharkhand | *Plasmodium* | 16.2% to 18.8% | - | - | - | - | 12.80% |
| Chandrashekar et al. (2019) | Karnataka (Mangaluru) | *Plasmodium* | 5% (5 cases = 2 Pv, 1 Pf, 2 Pf/Pv) | - | - | 29.5% (21 cases = 14 Pv, 3 Pf, 4 Pf/Pv) | - | 36.6% (26 cases = 18 Pv, 4 Pf, 4 Pf/Pv) |
| Palem and Pal (2019) | Karnataka (Mangaluru) | *Plasmodium* | 9.7% to 12.1% | - | 0 to 2.4% | - | - | 12.1% to 17% |
| Singh et al. (2020) | Madhya Pradesh (Maihar) | *Plasmodium* | - | - | - | - | - | 35.70% |
| Rao et al. (2021) | Andhra Pradesh (Visakhapatnam) | *Plasmodium* | 15% | - | - | 54% | 3% | 46% |

**Supplemental Table 5**. Details of studies on neonatal and congenital malaria in India

| **Authors** | **Title** | **Area** | ***n*** | **Gender** | **Age (days)** | **Mother** | **Type of delivery** | **Symptoms** | ***Plasmodium*** | **Treatment** | **Outcome** |
| --- | --- | --- | --- | --- | --- | --- | --- | --- | --- | --- | --- |
| Abraham (2015) | Congenital Malaria Mimicking Sepsis - A Case Report from Malaria Endemic Area in Central India | Uttar Pradesh | 1 | Male | 20 | Primigravidae | Vaginal | abdominal distension, fever, jaundice, lethargy, palor, poor feeding | *Pv* | IV ATB, IV Fluids, Oother supportive care, IV artesunate | Discharged |
| Bhatia and Bhatia (2019) | Congenital malaria due to Plasmodium vivax- A report of two cases | Rajasthan | 2 | Male | 6 | - | - | abdominal distension, fever, hepatosplenomegaly, jaundice, lethargy, palor, poor feeding | *Pv* | CQ (10 mg/Kg then 5 mg/Kg at 6, 24 and 48 hours) | Discharged |
|  |  |  |  | Female | 5 | - | - | abdominal distension, fever, hepatosplenomegaly, jaundice, lethargy, palor, poor feeding | *Pv* | CQ (10 mg/Kg then 5 mg/Kg at 6, 24 and 48 hours) | Discharged |
| Bhatia et al. (2016) | Congenital Malaria due to Plasmodium Vivax Infection in a Neonate | Rajasthan | 1 | Male | 6 | - | - | fever, hepatosplenomegaly, jaundice, lethargy, loose stools, palor, poor feeding/not feeding | *Pv* | IV ampicillin and gentamycin, then CQ | Discharged |
| Balatbat et al. (1995) | Congenital Malaria in a Nonidentical Twin | California | 1 | Female | 30 | - | Caesarian | fever, hepatosplenomegaly, lethargy, palor, poor feeding | *Pv* | IV ampicillin and cefotawime sodium, then CQ | Discharged |
| Chandelia et al. (2013) | Congenital vivax malaria: rare or underdiagnosed infection | Delhi | 1 | Male | 8 | - | - | fever, hepatosplenomegaly, jaundice, palor, poor feeding | *Pv* | IV ampicillin and amikacin, then CQ | Discharged |
| Gandhi et al. (2011) | Neonatal Plasmodium vivax malaria: an overlooked entity | Delhi | 1 | Male | 26 |  | Vaginal | abdominal distension, fever, hepatosplenomegaly, loose stools, palor | *Pv* | CQ (10 mg/Kg then 5 mg/Kg at 6, 24 and 48 hours) | Discharged |
| Gathwala et al. (2015) | Congenital Malaria with Atypical Presentation: A series of three case reports | Haryana | 3 | Male | 17 | Multigravidae | Vaginal | abdominal distension, fever, jaundice, hepatosplenomegaly, palor | *Pv* | CQ (10 mg/Kg then 5 mg/Kg at 6, 24 and 48 hours) | Discharged |
|  |  |  |  | Male | 21 | Primigravidae | Vaginal | abdominal distension, fever, jaundice, hepatosplenomegaly, palor | *Pv* | CQ (10 mg/Kg then 5 mg/Kg at 6, 24 and 48 hours) | Discharged |
|  |  |  |  | Male | 38 | Primigravidae | Vaginal | abdominal distension, fever, jaundice, hepatosplenomegaly, palor | *Pv* | Injectable ATB, then CQ + Blood transfusion | Discharged |
| Mohan and Maithani (2010) | Congenital Malaria due to Chloroquine-Resistant *Plasmodium* Vivax: A Case Report | Uttar Pradesh | 1 | Male | 26 | - | Caesarian | fever, hepatosplenomegaly, palor | *Pv* | IV ATB (ceftriaxn and amoikacin), IV Fluids, antipyretics, then CQ (25 mg/Kg, 20 mg/Kg, and hen 10 mg/Kg 8 hourly for 7 days) | Discharged |
| Choudhury and Das (2021) | Case series on congenital malaria from a tertiary care hospital in North Eastern India | West bengal | 5 | Male | 28 | - | Vaginal | Inconsolable cry, palor | *Pv* |  | Discharged |
|  |  |  |  | Male | 21 | - | Vaginal | Inconsolable cry, poor feeding | *Pv* |  | Discharged |
|  |  |  |  | Female | 56 | - | - | Palor, organomegaly | *Pv* |  | Discharged |
|  |  |  |  | Male | 21 | Primigravidae | Caesarian | Lethargy, poor feeding | *Pf* | IV ATB | Discharged |
|  |  |  |  | Male | 21 | Multigravidae | Vaginal | Jaundice | *Pv* |  | Discharged |
| Rai et al. (2015) | Congenital malaria in a neonate: case report with a comprehensive review on differential diagnosis, treatment and prevention in Indian perspective | Delhi |  | Male | 21 | Primigravidae | Vaginal | abdominal distension, fever, hepatosplenomegaly, palor, poor feeding, tachychardia | *Pv* | ATB, then IV artesunate | Discharged |
| Saha et al. (2009) | Congenital malaria due to Plasmodium vivax and Plasmodium falciparum in a neonate | Jharkhand | 1 | Female | 27 |  | Vaginal | cough, fever, hepatosplenomegaly, palor | *Pf* + *Pv* | CQ (25 mg/Kg for 3 days) + Blood transfusion | Discharged |
